# Determinants of viscoelasticity and flow activation energy in biomolecular condensates

**DOI:** 10.1126/sciadv.adi6539

**Published:** 2024-02-16

**Authors:** Ibraheem Alshareedah, Anurag Singh, Sean Yang, Vysakh Ramachandran, Alexander Quinn, Davit A. Potoyan, Priya R. Banerjee

**Affiliations:** ^1^ Department of Physics, University at Buffalo, Buffalo, NY 14260, USA.; ^2^ Department of Chemistry, Iowa State University, Ames, IA 50011, USA.

## Abstract

The form and function of biomolecular condensates are intimately linked to their material properties. Here, we integrate microrheology with molecular simulations to dissect the physical determinants of condensate fluid phase dynamics. By quantifying the timescales and energetics of network relaxation in a series of heterotypic viscoelastic condensates, we uncover distinctive roles of sticker motifs, binding energy, and chain length in dictating condensate dynamical properties. We find that the mechanical relaxation times of condensate-spanning networks are determined by both intermolecular interactions and chain length. We demonstrate, however, that the energy barrier for network reconfiguration, termed flow activation energy, is independent of chain length and only varies with the strengths of intermolecular interactions. Biomolecular diffusion in the dense phase depends on a complex interplay between viscoelasticity and flow activation energy. Our results illuminate distinctive roles of chain length and sequence-specific multivalent interactions underlying the complex material and transport properties of biomolecular condensates.

## Teaser

A complex interplay between multivalent interactions and chain length controls the dynamical properties of biomolecular condensates.

## INTRODUCTION

Biomolecular condensates are phase-separated intracellular granules harboring multiple proteins, nucleic acids, and other biomolecules and are ubiquitous in almost all living systems (*1*, *2*). They have been implicated in key biological processes including stress response (*3*, *4*), gene regulation (*5*), genome organization and maintenance (*6*), mitochondrial signaling processes (*7*), and intracellular storage (*8*). Further, aberrant condensates are thought to be involved in disease processes including neurodegenerative disorders and certain types of cancer (*9*–*14*). From an engineering standpoint, biomolecular condensates offer programmable and biocompatible self-assembled soft colloidal structures that have substantial potential as artificial organelles, in intracellular cargo delivery and controlled release, and in creating stimuli-responsive artificial cell–like entities (*15*–*20*). Therefore, understanding the fundamental physical properties of biomolecular condensates, such as their material properties and network structure (*21*) and how they are linked to the specific features of the component biopolymers, is an important active area of the current research in the field.

Recently, we and others have shown that reconstituted biomolecular condensates are complex fluids with condensate-spanning viscoelastic networks that are present in both homotypic condensates formed by a single protein component and heterotypic condensates formed by proteins and nucleic acids (*15*, *22*, *23*). Studies in live cells subsequently indicated that the nucleolus, an archetypal protein–nucleic acid condensate that is responsible for ribosomal RNA (rRNA) biogenesis and processing, displays viscoelastic behavior (*24*). The viscoelasticity of the nucleolus has been proposed to be an important physical determinant of its function in facilitating the outward flow of processed rRNA (*24*–*27*). In addition, it has also been suggested that liquid-to-solid transitions in some ribonucleoprotein condensates can lead to pathological outcomes (*12*, *28*–*30*). Collectively, these recent advances show that biological condensates have unique and complex material properties, which are likely to play key roles in dictating their biological functions and roles in disease processes. However, deciphering the origins of condensate viscoelastic properties remains challenging because of the complex dependence of these properties on many physicochemical factors including chain length, intermolecular interactions, and the structure of constituent proteins and nucleic acids (*26*, *31*–*34*).

To establish mechanistic links between biopolymer sequence and structure and the viscoelastic behaviors of condensates, here, we use a multiscale approach combining microrheology and molecular simulations. We use mixtures of Arg-Gly–rich intrinsically disordered polypeptides (RG-IDPs) and single-stranded DNA (ssDNA) as model systems that form viscoelastic condensates (*15*). Because of the modular design of the polypeptide and ssDNA components, these synthetic condensates are suitable for systematically exploring the distinct effects of sequence-encoded multivalent biomolecular interactions and the effect of polymer chain length on the condensate dynamical properties. First, we use optical tweezer–based microrheology (*15*) to measure the viscoelastic shear moduli and the relaxation timescales for the condensate-spanning networks of heterotypic RG-IDP–ssDNA condensates. In parallel, we perform temperature-controlled video particle tracking (VPT) to probe the activation energy for the network flow of these condensates, which represents the energy barrier for reconfiguration of the condensate fluid network. Our measurements reveal that increasing the strength of sticker motifs in RG-IDPs increases the energy barrier for network reconfiguration and the frequency-dependent viscoelasticity of these condensates in a correlative manner. Conversely, chain length variations only affect the viscoelasticity of these condensates without any notable change in the energy barriers to the reconfiguration of viscoelastic fluid networks. Atomistic simulations reveal that the flow activation energy of condensates is directly linked to the dissociation of sticker motifs from DNA chains, providing a microscopic origin of this apparently anomalous behavior. These findings are further corroborated by carrying out simulations of condensates with coarse-grained resolution of biomolecules, which closely mimic experiments. Coarse-grained simulations show that higher dissociation barriers of binary complexes give rise to denser condensates featuring higher critical temperatures and viscosities. By varying peptide chain length at a fixed sticker concentration, we further demonstrate that flow activation energy is primarily determined by the energetics of IDP sticker–DNA complexes and not by the polymer length. These results collectively suggest that flow activation energy is a unique reporter of the strength of multivalent intermolecular interactions within biomolecular condensates. We find that polypeptide diffusion in the dense phase is inversely correlated with the flow activation energy and is not substantially altered by the ssDNA length variation, even when such a variation leads to an order of magnitude change in the bulk viscosity of the condensates. These results suggest that biomolecular diffusion in the dense phase is determined by a complex interplay between viscosity and flow activation energy and indicate that intra-condensate transport is governed by a reaction-limited diffusion mechanism (*35*, *36*). Overall, our computational and experimental analyses of both material properties and the flow activation energy enable us to dissect the distinctive roles of chain length and sticker valence in viscoelasticity and macromolecular transport in biomolecular condensates.

## RESULTS

### Peptide-ssDNA condensates follow the Arrhenius law of viscosity and have a well-defined activation energy of viscous flow

The concept of biomolecular condensates being a viscoelastic network fluid (*21*) implies at least two measurable quantities: the network reconfiguration timescale, which is the timescale of the network flow, and the energy barrier for reconfiguration of the condensate-spanning fluid network, called activation energy of the network flow. Recently, laser tweezer–based microrheology has been used to quantify the timescale of condensate network flow (*15*, *22*, *23*); however, according to our knowledge, the flow activation energy has not been reported for any biomolecular condensates. To probe both of these quantities, we first used a designed heterotypic condensate system formed by multivalent RG-rich repeat polypeptide, [RGRGG]_5_, and a 40–nucleotide (nt)–long ssDNA, dT40 (fig. S1). Notably, these peptide-ssDNA condensates do not show any signs of physical aging over time (fig. S2). RG-IDPs have previously been shown to provide a modular platform to dissect the roles of sticker and spacer residues on the phase behavior and material properties of heterotypic IDP-RNA condensates (*15*, *31*). The sticker-spacer classification of associative polymers is based on recent works distinguishing amino acids that directly contribute to interchain interactions (stickers) and amino acids that modulate the solvation and structural features of the chain (spacers) (*37*–*42*). In the present context, arginine residues are defined as stickers because they enable nucleic acid binding through a hierarchy of electrostatic, cation-π, and π-π interactions (*31*, *32*, *43*).

**Fig. 1.**
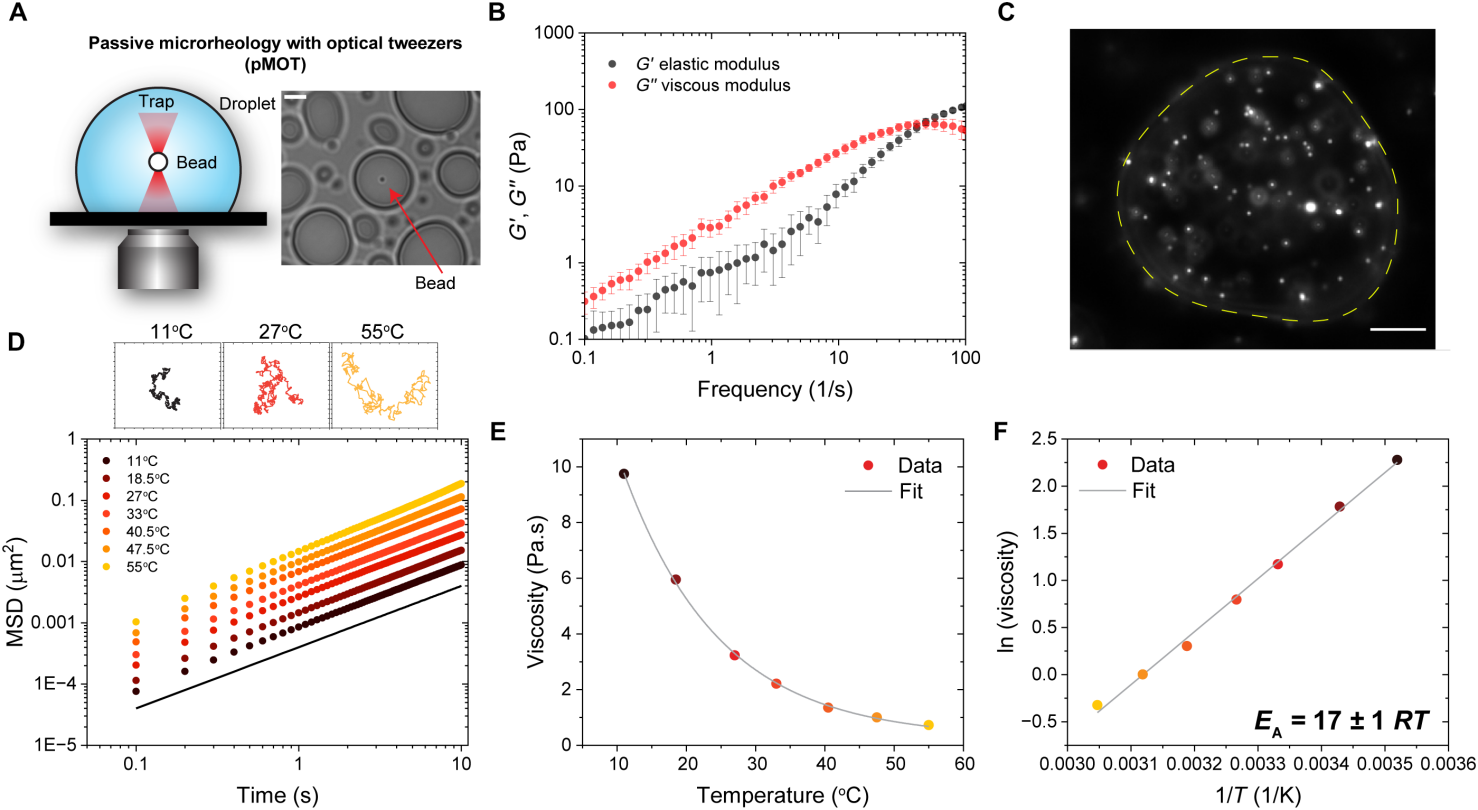
RG-repeat peptide–ssDNA condensates follow an Arrhenius law of viscosity. (**A**) Scheme showing the experimental setup for the passive pMOT experiments. (**B**) Average frequency-dependent viscoelastic moduli of [RGRGG]_5_-dT40 condensates (also see fig. S3). (**C**) Representative fluorescence image of 200-nm yellow-green fluorescent beads embedded within an [RGRGG]_5_-dT40 condensate. Scale bar, 10 μm. (**D**) Ensemble-averaged mean squared displacements (MSDs) of the 200-nm beads within [RGRGG]_5_-dT40 condensates at different temperatures. The black line has a slope that corresponds to a diffusivity exponent α = 1. The insets show representative particle trajectories at three different temperatures, as indicated. (**E**) Viscosity of [RGRGG]_5_-dT40 condensates plotted against temperature. The gray line is an exponential decay fit using Eq. 1 added to a constant. (**F**) Arrhenius plot of viscosity and temperature. The gray line represents a linear fit. Activation energy *E*_A_ is calculated from the slope of the line according to Eq. 2.

We used our passive microrheology with optical tweezers (pMOT) assay (*15*, *44*) and measured the rheological moduli of IDP-ssDNA condensates formed by mixing [RGRGG]_5_ and dT40 in a buffer containing 25 mM MOPS (pH 7.5), 25 mM NaCl, and 20 mM dithiothreitol (Fig. 1A and figs. S1 and S3). We found that [RGRGG]_5_-dT40 condensates exhibit viscoelastic behavior similar to a Maxwell fluid that features a single crossover frequency between a dominant viscous regime at low frequencies and a dominant elastic regime at high frequencies (Fig. 1B). The terminal relaxation time is found to be ~20 ms, and the terminal condensate viscosity is 3.3 ± 0.2 Pa.s (at *T* = 27°C; Fig. 1B). The terminal relaxation time represents the longest relaxation time of the network (defined as the inverse of the crossover frequency), while the terminal viscosity is the zero-shear viscosity representing the dominant viscous behavior at long timescales (*15*). We next probed the flow activation energy by evaluating the temperature dependence of the terminal viscosity of these condensates. To this end, we used temperature-controlled VPT to measure the terminal viscosity of [RGRGG]_5_-dT40 condensates as a function of temperature ranging from ~10° to 70°C (Fig. 1, C and D, and fig. S4). We chose an acquisition rate (100 ms) that is larger than the terminal relaxation time (~20 ms) of the condensate to probe the region of timescales where the viscous modulus dominates the material response. Further, the chosen temperature range is well below the upper cloud point temperature of these condensates (>90°C) (fig. S5) (*15*). Our VPT measurements revealed that increasing temperature leads to a decrease in the viscosity to ~0.7 Pa.s at 55°C. Similarly, decreasing temperature led to an increase in viscosity (~10 Pa.s at 11°C) of the same condensates. The viscosity variation with temperature can be fitted with an Arrhenius-like exponential decay function (Fig. 1E and fig. S4)

$$\eta =\eta_{0}\text{exp}\left(\frac{E_{A}}{\mathrm{RT}}\right)$$ (1)

where *T* is the temperature in kelvin, *R* is the universal gas constant, and *E*_A_ is the energy barrier for reconfiguration of the condensate fluid network. The viscosity prefactor η_0_ depends on local molecular packing density and is often related to the frequency of barrier-crossing attempts in rate theories (*45*). This exponential scaling of viscosity with temperature is in accordance with the Arrhenius behavior observed for liquids above the glass transition temperatures where the molecular dynamics are dominated by rare events involving jump-like molecular reconfiguration (*46*–*48*). We note that for complex fluids, Arrhenius-like behavior often masks complex molecular rearrangements taking place through several steps (*49*). Plotting the natural log of viscosity against the inverse of temperature yields a linear relation with a slope equal to *E_A_*/*R*

$$\text{ln}\;\eta =\text{ln}\;\eta_{0}+\frac{E_{A}}{R}\left(\frac{1}{T}\right)$$ (2)

Figure 1F shows that [RGRGG]_5_-dT40 condensates obey the Arrhenius law (also see fig. S4). The resulting activation energy as extracted from the fit was 17 ± 1 *RT* (at *T* = 25°C), which is equivalent to ~42 kJ/mol. This is comparable to the flow activation energy of 90% glycerol/water mixture (fig. S6) (*50*, *51*). This indicates that the energy barrier for the condensate network to reconfigure is ~17 times the thermal energy of the system in the standard conditions, as per the theory of rate processes (*47*). The observation that the temperature dependence of viscosity for [RGRGG]_5_-dT40 condensate obeys an Arrhenius relation also indicates that these condensates are thermo-rheologically simple fluids within the limits of our experimental temperature range, meaning that they obey the time-temperature superposition principle (*52*).

The results discussed above were obtained using peptide-ssDNA condensates formed at a 1:1 mass ratio. To further test whether the activation energy depends on the bulk mixture composition, we performed experiments with condensates formed at two additional mass ratios of 0.5 and 1.5 of dT40/[RGRGG]_5_. Our measurements show that the bulk viscosity and the activation energy are independent of the mixture composition for the same peptide-ssDNA condensate (fig. S7). This observation is consistent with our previous measurements where we showed that the dynamical properties of the dense phase remain insensitive to the mixture composition of a similar condensate-forming system (*53*).

### The flow activation energy and viscoelastic properties of heterotypic peptide–ssDNA condensates are governed by sequence-dependent intermolecular interactions

The theory of rate processes by Eyring (*47*) suggests that the flow activation energy of a fluid depends on the enthalpy of interactions among the molecular components. For a fluid to flow, the molecules have to rearrange to accommodate the exchange of molecules in space (fig. S8). Analogous to a chemical reaction, the reconfiguration of a fluid network has an energy barrier that is termed the activation energy of viscous flow (fig. S8) (*47*). When the temperature is increased, the rate of molecules crossing the activation energy barrier increases, leading to a faster flow. Furthermore, when a shear force is applied, the symmetry of the energy barrier is broken such that the rate of molecules crossing the activation barrier in the direction parallel to the flow is higher than those crossing the activation barrier in the opposite direction (fig. S8) (*47*).

**Fig. 2.**
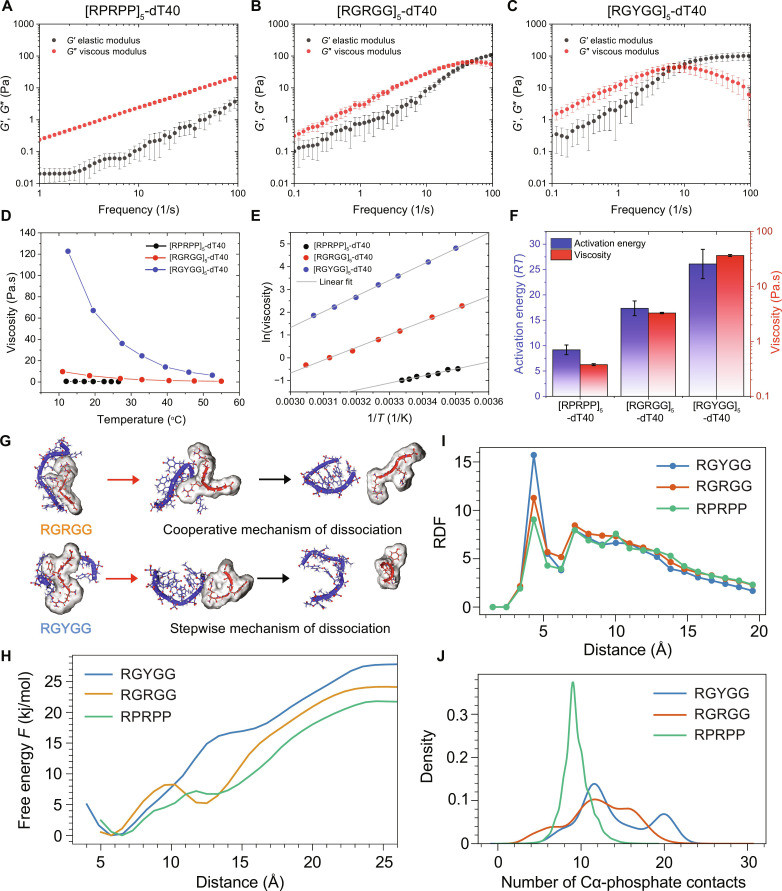
Sequence-encoded intermolecular interactions govern the viscoelastic behavior and flow activation energy of peptide-ssDNA condensates. (**A** to **C**) Frequency-dependent average viscoelastic moduli of [RPRPP]_5_, [RGRGG]_5_, and [RGYGG]_5_ condensates with dT40, respectively. Error bars represent the standard deviation estimated from ~40 datasets. (**D**) Viscosity variation with temperature for peptide-dT40 condensates. The peptides tested are [RGRGG]_5_, [RPRPP]_5_, and [RGYGG]_5_. (**E**) Arrhenius plots of viscosity versus temperature for the three peptide sequences in (D). (**F**) Variation of viscosity (at *T* = 27°C; red) and flow activation energy (blue) for [RPRPP]_5_, [RGRGG]_5_, and [RGYGG]_5_ condensates with dT40. (**G**) Snapshot of molecular configurations from umbrella sampling simulations of peptide-ssDNA dissociation. Shown here are two distinct dissociation profiles observed, which are termed stepwise in case of RGRGG and RPRPP motifs due to the flexibility of the chains and cooperative for RGYGG due to the relatively rigid orientation of the chains in the complex. (**H**) Free energy as a function of the center of mass distance between ssDNA and peptides computed from umbrella sampling simulations. (**I**) Radial distribution functions computed from multichain simulations showing higher affinity of RGYGG peptide to cluster around ssDNA relative to RGRGG and RPRPP peptides. (**J**) Probability density of contacts quantified by distance threshold of phosphate and peptide backbone distances for different peptide-ssDNA complexes.

Several recent studies have reported that the physical properties of biomolecular condensates are sensitively dependent on the constituent protein and nucleic acid primary sequence composition and patterning (*18*, *37*, *54*–*59*). We therefore asked how the polypeptide sequence features affect the energy barrier for flow. To address this question, we used three nucleic acid–binding repeat IDPs with variable sticker motifs. Specifically, we used a variant of [RGRGG]_5_, [RGYGG]_5_, which has been shown to exhibit higher upper critical solution temperature for phase separation, and its condensates with RNA have been shown to exhibit greater viscoelastic response (fig. S5) (*15*). We next used a variant with proline spacers [RPRPP]_5_ that was suggested to bind RNA with weaker affinity (fig. S5) (*15*). We first performed microrheology experiments on individual condensates formed by these three peptides ([RGRGG]_5_, [RGYGG]_5_, and [RPRPP]_5_) with a 40-nt ssDNA, dT40 (Fig. 2, A to C), and observed that the rank order of condensate viscoelastic properties is [RGYGG]_5_-dT40 > [RGRGG]_5_-dT40 > [RPRPP]-dT40 (Fig. 2, A to C, and fig. S9A). Through VPT experiments, we next measured the terminal viscosity of these condensates at *T* = 27°C. Consistent with our laser tweezer–based microrheology experiments, we find that [RPRPP]_5_-dT40 condensates have the lowest viscosity of 0.38 ± 0.02 Pa.s, which is followed by [RGRGG]-dT40 condensates (3.28 ± 0.09 Pa.s), whereas the [RGYGG]_5_-dT40 condensates registered the highest viscosity (37± 2 Pa.s) (fig. S9B). Next, to measure the flow activation energy of these peptide-ssDNA condensates, we performed temperature-controlled VPT experiments (Fig. 2, D to F, and fig. S9, C to F). We find that [RGYGG]_5_-dT40 condensates have an activation energy of 26 ± 3 *RT*, which is substantially greater than [RGRGG]_5_-dT40 condensates that have an activation energy of 17 ± 1 *RT* (Fig. 2, D to F, and fig. S9). Further, [RPRPP]_5_-dT40 condensates featuring weakened nucleic acid binding by the peptide exhibited substantially lower activation energy (9 ± 1 *RT*; Fig. 2, D to F; fig. S9; and table S1). To further check whether the viscoelasticity and flow activation energy change in a correlated manner for the peptide-dT40 condensates, we compared the activation energy of these condensates with their respective terminal relaxation time, estimated from the *G*′, *G*″ crossover frequency (Fig. 2, A to C, and fig. S10A) and elastic modulus (*G*′) at 10 Hz (fig. S10B).

To shed light on the nature of intermolecular interactions that give rise to the observed differences in flow activation energy in these condensates, we used explicit solvent all-atom molecular dynamics simulations of peptide-ssDNA chains. In our simulations, we used single penta-residue peptides RGRGG, RGYGG, and RPRPP interacting with a poly(dT) DNA of length 8. First, through umbrella sampling simulations, we mapped the free energy of dissociation as a function of the intermolecular center of mass distance (Fig. 2H). The free energy profiles reveal a two-state dissociation mechanism (Fig. 2G), with different barrier heights separating the bound state from the first intermediate configuration. We hypothesize that the combination of first and second barriers is linked with the experimentally observed flow activation energy. This is motivated by the fact that shearing of condensate is inevitably accompanied by the breaking and making of contacts between the peptide and ssDNA molecules. Consistent with experimentally measured activation energies, we find that RGYGG peptide has a substantially higher dissociation free energy compared to the RGRGG and RPRPP peptides. Furthermore, we observe that the dissociation happens in a stepwise manner for RGRGG and RPRPP peptides due to the greater flexibility of arginine stickers. On the other hand, the mechanism of dissociation appears to be more cooperative for RGYGG, which necessitates the breaking of a substantial number of contacts to arrive at the first intermediate state (Fig. 2H). To test whether a similar dissociation profile holds for multichain systems, we next carried out enhanced sampling via simulated tempering simulations with a temperature range of 300 to 450 K on a system consisting of 12 peptide-ssDNA chains with 45 mg/ml biomolecular density. The radial distribution functions and the number of contacts between phosphate and peptide backbone groups (Fig. 2, I and J) provide further demonstration that RGYGG motifs have a higher affinity for ssDNA both at the level of single chains and also within a condensate microenvironment relative to RGRGG and RPRPP motifs. We note, however, that dissociation-free energies still cannot be directly compared to the flow activation energy, because the effect of condensate environment and crowding will likely affect the dissociation events taking place during shearing of condensates. Therefore, we have carried out simulations using coarse-grained models (Fig. 5), which allow us to simulate condensates at different temperatures and extract flow activation energy directly from temperature-dependent viscosity profiles. Together, our experimental and computational analyses quantitatively reveal that the strength of intermolecular peptide–ssDNA interactions is a key determinant of the flow activation energy and the viscoelasticity of these condensates.

### Chain-length variation has a differential effect on the viscoelasticity and flow activation energy of peptide-ssDNA condensates

**Fig. 3.**
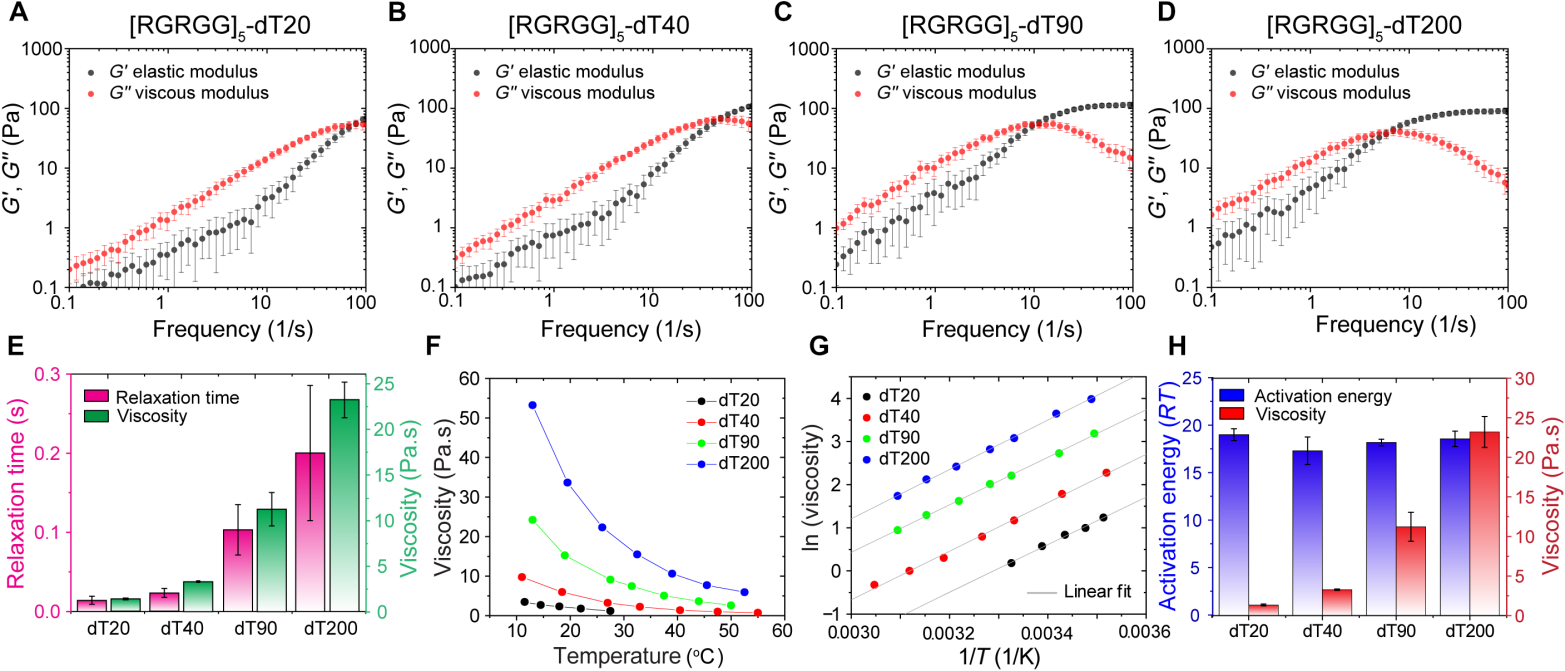
ssDNA length alters the viscoelasticity of peptide-ssDNA condensates. (**A** to **D**) Average frequency-dependent viscoelastic moduli of [RGRGG]_5_-dT*n* condensates, where *n* is 20, 40, 90, and 200, respectively. (**E**) Terminal relaxation times (pink) and VPT-measured viscosity (green) of [RGRGG]_5_-dT*n* condensates corresponding to (A) to (D) at *T* = 27°C. (**F**) Viscosity variation with temperature for [RGRGG]_5_-dT*n* condensates, where *n* ranges from 20 to 200. (**G**) Arrhenius plots for [RGRGG]_5_-dT*n* condensates. Note the similar slopes for all DNA lengths. Gray lines are linear fits to the data. (**H**) Flow activation energy and viscosity (at 27°C) of [RGRGG]_5_-dT*n* condensates. Activation energy *E_A_* and the preexponential factor (fig. S13) are calculated from the slope and intercept of the linear fit according to Eq. 2.

In addition to the strength of interchain interactions, the viscoelastic response of complex fluids can also be tuned by the length of associative polymer chains (*60*). We tested this idea by probing the effect of ssDNA length on the rheology of peptide-ssDNA condensates. We used poly(dT) sequences featuring different numbers of nucleotides ranging from 20 to 200 nt (dT20, dT40, dT90, and dT200). Microrheology experiments on condensates formed by [RGRGG]_5_ with ssDNA of variable length at identical mass concentrations (5 mg/ml peptide and 5 mg/ml ssDNA) showed an increased viscoelastic response with increasing ssDNA length. For instance, [RGRGG]_5_-dT20 condensates showed a dominant viscous behavior across the experimentally accessible frequency range, with a terminal relaxation time of ~14 ms (Fig. 3, A and E, and fig. S11A) and a terminal viscosity of 1.3 ± 0.1 Pa.s (at 27°C; Fig. 3E and fig. S11B). Increasing the DNA length to 40 nt led to a higher viscosity (3.28 ± 0.09 Pa.s) and a longer terminal relaxation time (~20 ms; Fig. 3, B and E). Further lengthening of the DNA chain to 90 nt (dT90) led to an increase in viscosity to 11 ± 2 Pa.s and an increase in relaxation time to ~100 ms (Fig. 3, C and E). Last, condensates formed by dT200 showed a stronger viscoelastic behavior with a viscosity of 23 ± 2 Pa.s and a terminal relaxation time of ~200 ms, which is more than an order of magnitude higher as compared to the condensates formed by dT20 (Fig. 3, D and E). These results establish an important role of ssDNA length in dictating the viscoelastic properties of these condensates (Fig. 3, A to E, and fig. S11, A and B).

Next, to probe the impact of the polymer length on the energy barrier of network reconfiguration, we measured their flow activation energy as a function of ssDNA length. Although the condensate viscosity increased by an order of magnitude with increasing ssDNA length from 20 to 200 nt, temperature-controlled VPT measurements reveal similar exponential scaling of condensate viscosity with temperature (Fig. 3F and fig. S11, C and D). Intriguingly, the Arrhenius plots for peptide-ssDNA condensates showed similar slopes irrespective of the ssDNA length (20, 40, 90, and 200 nt), indicating a constant activation energy (Fig. 3G; fig. S11, E to H; and table S1). This means that the flow activation energy of these condensates does not change with increasing DNA length (from 20 to 200 nt) despite an order of magnitude change in condensate viscoelasticity (Fig. 3H).

**Fig. 4.**
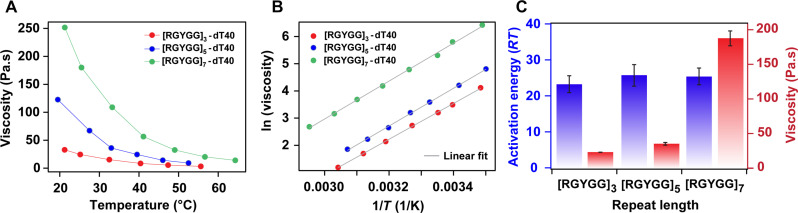
Peptide repeat length controls condensate viscosity without affecting the flow activation energy. (**A**) Viscosity variation with temperature for [RGYGG]*_n_*-dT40 condensates, where *n* is 3, 5, and 7. (**B**) Arrhenius plots for [RGYGG]*_n_*-dT40 condensates. Gray lines are linear fits to the data according to Eq. 2. (**C**) Flow activation energy and viscosity (at 27°C) of [RGYGG]*_n_*-dT40 condensates.

According to Eyring’s transition state theory, the flow activation energy, which is the energy barrier of reconfiguring the fluid network, is predominantly determined by the intermolecular interactions between the polymer chain segments (fig. S8) (*47*). On the basis of this argument, we posited that the flow activation energy will also remain invariant as the peptide repeat length is increased without altering the sticker motifs. To test this idea, we performed thermorheological measurements on the peptide [RGYGG]*_n_* and ssDNA dT40. The peptide repeat number *n* was varied between 3, 5, and 7 in our experiments, keeping the mass ratio of the peptide and ssDNA as 1:1, which assured the unchanged concentrations of sticker motifs. The viscosities of the [RGYGG]*_n_*-dT40 condensates for *n* = 3, 5, and 7 at *T* = 27°C were 24 ± 1 Pa.s, 37 ± 2 Pa.s, and 185 ± 11 Pa.s, respectively. In addition, we found that longer peptides formed condensates with notably higher viscosity values at all tested temperatures (Fig. 4A; fig. S12, A and B; and table S1). However, our thermorheological analysis showed that condensates formed by repeat peptides with variable lengths in the presence of dT40 have similar flow activation energy (Fig. 4, B and C, and fig. S12, C and D). The estimated activation energies for the [RGYGG]*_n_*-dT40 condensates for *n* = 3, 5, and 7 are 24 ± 3 *RT*, 26 ± 3 *RT*, and 25 ± 2 *RT*, respectively. These results confirm that the flow activation energy is primarily controlled by the strength of intermolecular interactions between the sticker motifs and ssDNA and is insensitive to chain length.

### Interplay between intermolecular interactions and chain length controls the phase behavior, flow activation energy, and viscoelasticity of protein-ssDNA condensates

**Fig. 5.**
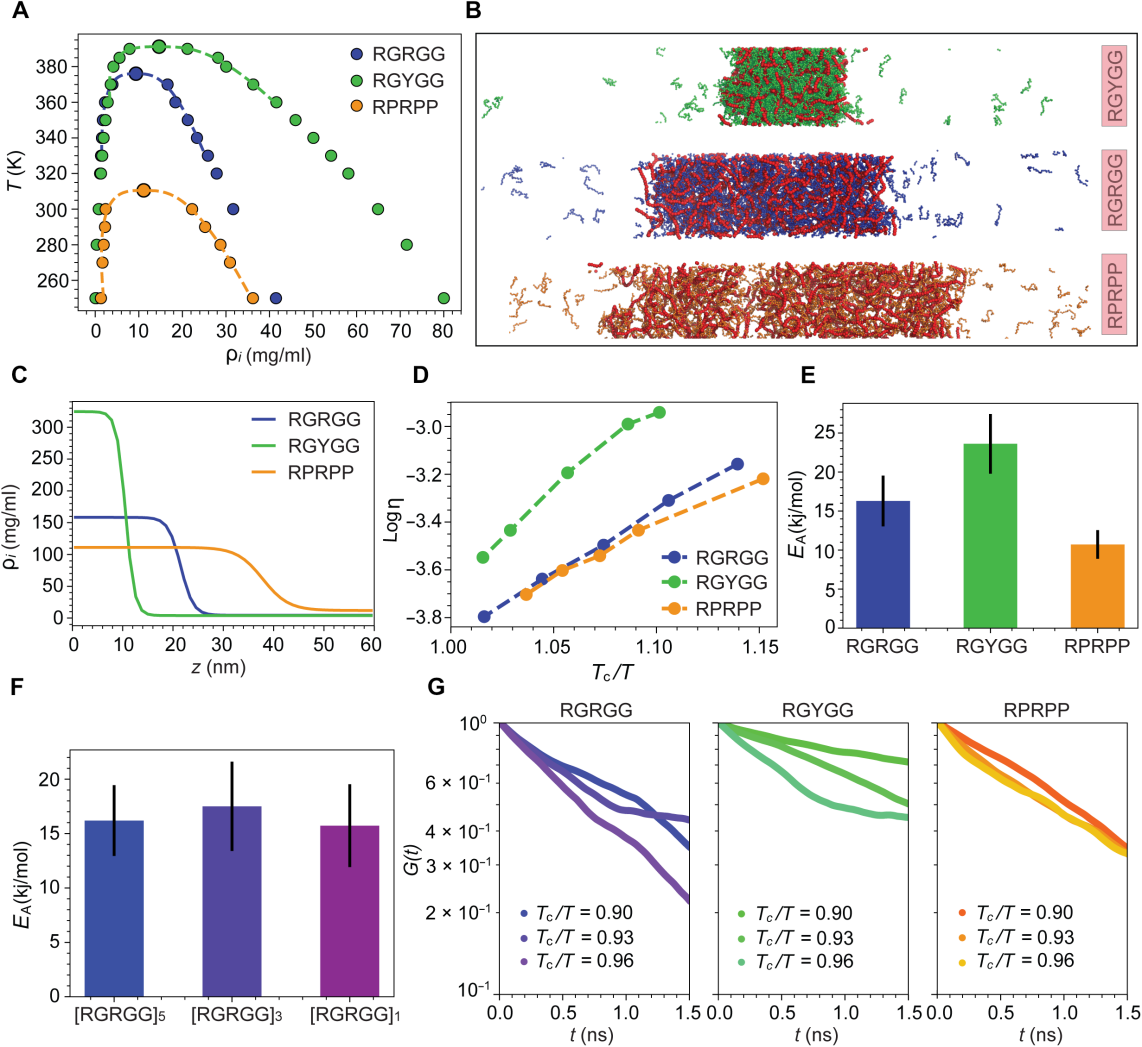
Quantifying the RG-IDP–ssDNA condensate material properties via coarse-grained molecular dynamics simulations. (**A**) Phase diagram of peptide-ssDNA condensates in the space of temperature and biomolecular density obtained from direct coexistence simulations. (**B**) Representative configurations from the direct coexistence simulations done at temperature *T* = 300 K for the three peptide-ssDNA systems. (**C**) Density profiles for the simulation boxes in (B). (**D**) Arrhenius plots for the three different peptide-ssDNA condensates extracted from the temperature-dependent viscosity profiles. (**E**) Activation energies extracted from Arrhenius plots for different peptides. (**F**) Activation energies extracted from Arrhenius plots for different repeat lengths of RGRGG (also see fig. S14). (**G**) Normalized relaxation modulus computed from ssDNA Rouse mode autocorrelation function for three different peptide-ssDNA systems showing different relaxation timescales of ssDNA chains surrounded by different sticker motifs.

To explore how sticker motifs in peptides and chain length affect the viscoelastic and phase behaviors of condensates, we carried out coarse-grained molecular dynamics simulations using full-length peptide and ssDNA chains at a similar mass ratio to our experimental conditions. In these simulations, we used a one bead per residue and one bead per nucleotide coarse-grained representation to carry out direct coexistence simulations with anisotropic box geometry and liquid condensate simulations with isotropic cubic box geometry (Fig. 5; also see Supplementary Materials and Methods for details) (*56*, *61*). For short-range protein-protein and protein-RNA interactions, we have used the hydrophobicity scale named CALVADOS2 obtained from Bayesian optimization of hydrophobicity parameters against gyration data with short-range cutoff distances (*62*). From direct coexistence simulations, we mapped the phase diagram of condensates as a function of biomolecular density and temperature (Fig. 5A). The critical temperature follows a trend that is consistent with our all-atom simulations (Fig. 2), whereby RGYGG motifs are stronger binders followed by RGRGG and RPRPP. This trend was also observed in earlier experimental results of these peptides with RNA (fig. S5). We note that both spacer and sticker residues contribute substantially to the stability of condensates because of vastly different hydropathy index ratios for G/P being 1.88 and for Y/R being 1.37, respectively. The molecular configurations obtained from simulations show that at the same temperature, RGYGG peptides create denser liquid slabs compared to RGRGG and RPRPP peptides (Fig. 5, B and C). Using the density of the condensed phase for each temperature obtained in direct coexistence simulations, we subsequently run multiple simulations of the condensed phase and computed viscosities using Green-Kubo relation (*63*). Arrhenius plots of viscosity variation with temperature show fairly robust linear trends for all the tested systems (Fig. 5D). The slopes of these linear trends, which quantify the flow activation energy, are positively correlated with the critical temperatures for phase separation (Fig. 5E). This shows that energetics of interchain interaction are one of the primary determinants of phase behavior, viscosity, and the flow activation energy of condensates. Consistent with all-atom simulations and experiments, we find that RGYGG motifs have higher activation energy due to higher sticker dissociation barriers shaped by an interplay of sticker and spacer motifs. To further test that sticker identity and concentration are the determining factors for the condensate flow activation energy independent of chain length, we carried out additional simulations with varying peptide lengths for the (RGRGG)*_n_* peptide with *n* = 1, 3, and 5. We find that activation energy remains comparable for all the systems, thereby providing further support to our mechanistic hypothesis that the binding energy landscape of ssDNA-peptide complexes, shaped by the sticker and spacer motifs, is the determining factor for the flow activation energy (Fig. 5F and fig. S14). Last, to gain a deeper insight into the viscoelastic nature of condensates, we have computed relaxation modulus by computing Rouse relaxation modes for equally sized ssDNA chains in the presence of our three peptides RGRGG, RGYGG, and RPRPP. The sticky Rouse model for associative polymers (*64*, *65*) suggests that the relaxation of a polymer chain is dictated by two distinct sets of relaxation modes: (i) a set of slow dissociation of stickers and (ii) a set of fast relaxation modes due to intrinsic relaxation of chains that are likely to be unaffected by associative interactions. By computing the first few of the slow modes, we find that ssDNA chains relax on different timescales in the presence of the three different peptides within condensates (Fig. 5G). Therefore, it is clear that sticker dissociation barriers from peptides chains, regardless of their length, have a strong capacity to retard the motions of longer DNA chains in the condensates, thereby leading to a substantially different viscoelastic behavior of the dense phase. Overall, our coarse-grained simulations shed light on the effect of peptide sequence and length on determining the flow activation energy and viscoelastic properties of ternary condensates, stressing the fact that intermolecular chain contacts are one of the primary determinants of the flow activation energy of these condensates.

### Polypeptide diffusion in the dense phase is inversely correlated with the flow activation energy

A key relevance of quantifying the material properties of biomolecular condensates lies in understanding how macromolecular transport is regulated in the dense phase. It is generally accepted that biomolecular diffusion reports on the material state of the condensates, with slower diffusion indicating higher viscosity of the dense phase. The perceived correspondence between molecular diffusion and material properties is one of the core principles used to infer the material states of condensates using fluorescence recovery after photobleaching (FRAP) (*66*). This correlation is based upon the assumption that the Stokes-Einstein equation, or some version of it, is applicable to model biomolecular diffusion within these condensates. However, transport properties of associative biopolymers, in theory, can be affected by reaction-diffusion mechanism rather than pure diffusion within a biomolecular condensate, especially when one considers the reversible association and dissociation of the chain with the condensate viscoelastic network (*66*). In addition, the viscoelastic network structure can result in a length scale–dependent molecular transport in the dense phase (*67*, *68*). Our microrheology results revealing distinct thermodynamic forces to tune condensate material properties prompted us to ask how intermolecular interactions and polymer chain length regulate biomolecular diffusion in the dense phase.

**Fig. 6.**
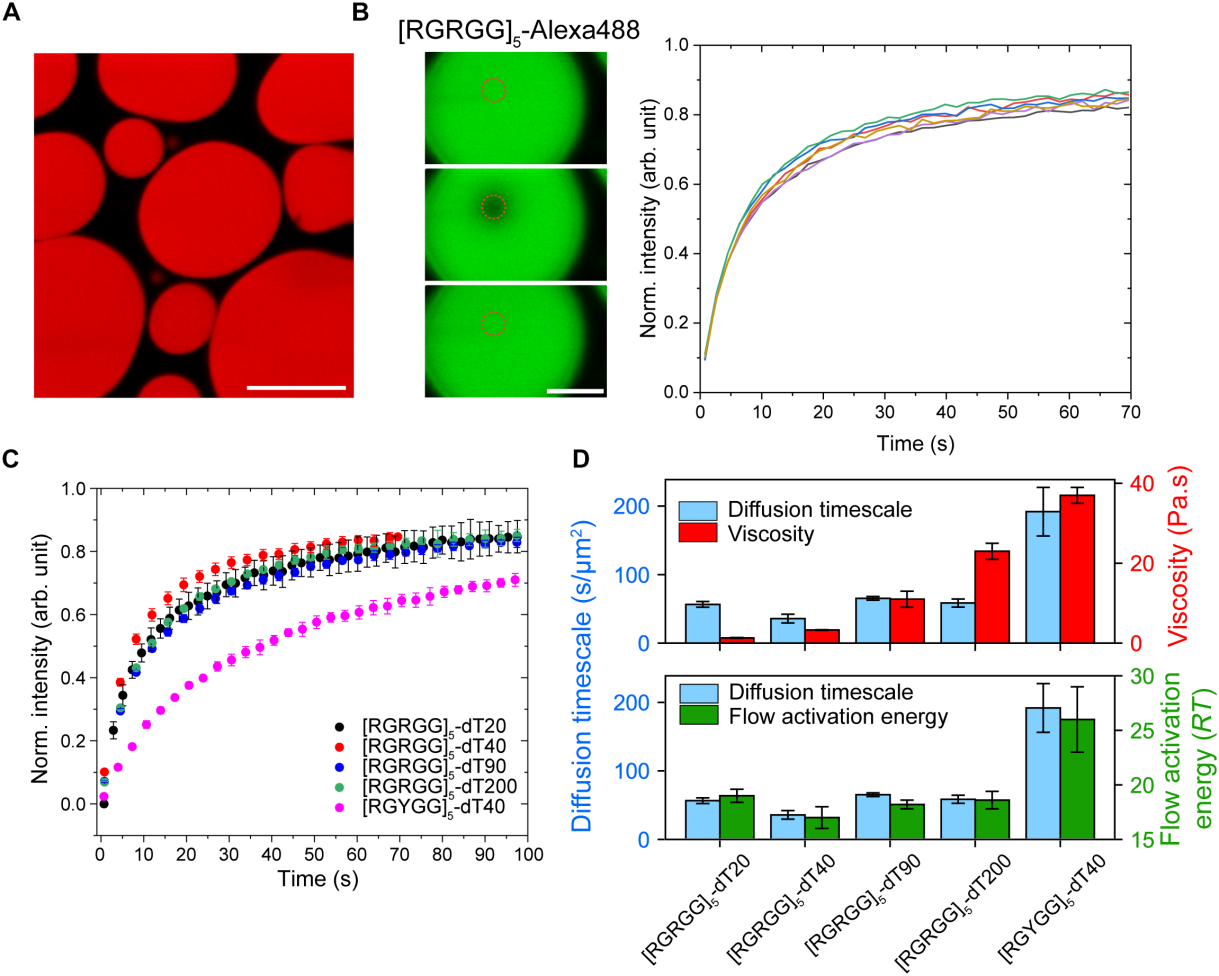
Polypeptide diffusion within peptide-ssDNA condensates scales with flow activation energy but not with the bulk viscosity of the dense phase. (**A**) Representative fluorescent image of [RGRGG]_5_-dT40 condensates visualized by Cy5-labeled dT40. Scale bar, 10 μm. (**B**) FRAP measurements for the Alexa488-labeled [RGRGG]_5_ peptide in [RGRGG]_5_-dT40 condensates. Intensity traces from six different condensates are shown. (**C**) Average FRAP intensity trace for Alexa488-labeled peptide in condensates formed by [RGRGG]_5_ and dT20, dT40, dT90, and dT200, as well as condensates formed by [RGYGG]_5_ and dT40, respectively. The value of the intensity is an average of four to six trials, and the error is the standard deviation of the same. (**D**) Diffusion timescale of Alexa488-labeled peptides in various peptide-ssDNA condensates as calculated from (C). The viscosity of the condensates (red, top) at *T* = 27°C and the flow activation energy of the same condensates (green, bottom) are also plotted. All samples were prepared at 5 mg/ml peptide and 5 mg/ml ssDNA in a buffer containing 25 mM MOPS (pH 7.5), 25 mM NaCl, and 20 mM dithiothreitol.

To probe the correlation between the viscoelastic properties and biomolecular transport, we chose three condensate systems. Our reference condensate is [RGRGG]_5_-dT40, which has a bulk viscosity of 3.28 ± 0.09 Pa.s and a flow activation energy of 17 ± 1 *RT*. The first variant system is [RGYGG]_5_-dT40 condensate, which has an ~10-fold higher viscosity (37 ± 2 Pa.s) and a higher activation energy of 26 ± 3 *RT*. This system is representative of a condensate with stronger sticker motifs (Fig. 2). The second variant system is [RGRGG]_5_-dT200, which has a significantly higher viscosity of 23 ± 2 Pa.s than the reference condensate but a similar activation energy of 18.6 ± 0.8 *RT*. This variant represents a condensate with enhanced viscoelasticity through chain length variation (Fig. 3). To estimate the diffusion timescale of polypeptides, we performed FRAP experiments using fluorescently labeled polypeptides under identical experimental conditions (Fig. 6, A and B; see Materials and Methods). In all condensates tested, near-complete FRAP recovery is observed. We calculated the apparent diffusion timescale (τ_D_) of the peptides as the FRAP recovery half-time normalized with respect to the radius of the bleaching area ( $\tau_{D}=\tau_{\text{FRAP}}/R_{\text{bleach}}^{2}$ ) (*69*–*71*). We find that [RGRGG]_5_-dT40 condensates have a diffusion timescale of τ_D_ = 36 ± 6 s/μm^2^ for the peptide ([RGRGG]_5_). For [RGYGG]_5_-dT40 condensates, the peptide dynamics are significantly slowed down, with an [RGYGG]_5_ peptide diffusion timescale of τ_D_ = 190 ± 30 s/μm^2^ (Fig. 6, C and D). This is not surprising given that [RGYGG]_5_-dT40 condensates have a higher bulk viscosity than [RGRGG]_5_-dT40. Contrastingly, when changing the DNA length ([RGRGG]_5_-dT200), the [RGRGG]_5_ peptide diffusion was unaltered, as evidenced by an almost identical FRAP recovery pattern and a similar diffusion timescale (59 ± 6 s/μm^2^; Fig. 6, C and D). The FRAP recovery traces of the [RGRGG]_5_ peptides appear almost insensitive to varying ssDNA length from 20 to 200 nt (Fig. 6, C and D), although the bulk viscosity changes by almost an order of magnitude (Figs. 3 and 4) with such variation (τ_D_ is 56 ± 4, 36 ± 6, 65 ± 3, and 59 ± 6 s/μm^2^ for dT20, dT40, dT90, and dT200 condensates, respectively). These results indicate that bulk viscosity does not govern polypeptide diffusion within these condensates (Fig. 6D, top), which would be expected if the Stokes-Einstein relation and Fick’s law of diffusion hold true for these condensates. Instead, our results demonstrate that there is a positive correlation between the flow activation energy and the peptide diffusion timescale within peptide-ssDNA condensates (Fig. 6D, bottom). This observation indicates that the observed diffusion dynamics of peptides are likely to be dominated by a reaction-diffusion mechanism where the strengths of peptide-DNA interactions regulate macromolecular transport in the dense phase in conjunction with condensate viscosity. Further, the FRAP recovery traces for Cy5-dT40 in [RGYGG]*_n_*-dT40 condensates for *n* = 3, 5, and 7, which reports the diffusion of the larger scaffolding component within the condensate microenvironment, revealed only partial recovery that decreases with increasing valence of the repeat peptide. These data suggest that the mobility of dT40 is strongly impeded by the condensate-spanning viscoelastic network of these condensates (fig. S15). Together, our data showcase deviations of transport properties from a purely diffusion-based mechanism. Given these observed complexities, molecular mobilities may not always be true reporters of the material properties of biomolecular condensates.

## DISCUSSION

The quantification of material properties of biomolecular condensates is gaining increasing attention because of their potential biological, therapeutic, and synthetic implications (*72*). A hallmark of numerous biomolecular condensates is the viscoelastic behavior manifesting in a dominant elastic response at short timescales and a dominant viscous response at longer timescales (*15*, *22*–*24*, *39*, *73*). The origin of viscoelasticity lies in the polymer nature of condensate components coupled with system-specific percolation transitions driven by associative interactions in these systems (*21*, *74*). Previously, we demonstrated a programmable nature of viscoelasticity of heterotypic condensates formed by RNA binding repeat polypeptides through peptide sequence design (*15*). Simultaneously, several pieces of evidence emerged demonstrating that viscoelasticity may be a common trait in both heterotypic and homotypic condensates (*22*, *23*, *73*). In a recent report, RNA entanglement was proposed to be the cause of the viscoelastic behavior of the nucleolus (*24*). In a separate set of studies, macromolecular regulators, such as molecular crowders, have been shown to substantially affect the phase equilibrium and material properties of condensates (*75*, *76*). These advancements point to a complex interplay between intermolecular interactions and polymer effects, such as protein and/or nucleic acid chain length, in governing the viscoelastic behavior of biomolecular condensates (*24*). Therefore, dissecting the distinct roles of intermolecular interactions and chain length is essential for understanding the molecular origin of condensate viscoelasticity as well as for devising suitable strategies to tune them. In this work, we have introduced a multiparametric approach to probe condensate viscoelasticity by combining microrheology measurements with thermo-rheological analysis in a series of designed peptide-ssDNA condensates. Furthermore, to shed light on the microscopic origins of the sequence- and length-dependent condensate material properties, we have carried out detailed multiscale molecular dynamics simulations with all-atom and coarse-grained models of condensates.

First, we showed that heterotypic condensates formed by oppositely charged macromolecules display Arrhenius-like temperature dependence of viscosity (*46*, *47*, *77*). This led us to quantify the condensate flow activation energy, which ranges from 9 to 26 *RT*. These values are within the same range as previously reported flow activation energies for synthetic complex coacervates using bulk rheology (*78*–*82*). Altering intermolecular interactions through sequence variation of the peptide leads to a correlative change in the flow activation energy, indicating that the energy barrier of the condensate network reconfiguration is primarily governed by the energetic barrier of peptide-DNA dissociation. We find that both stickers and spacers contribute to the free energy of dissociation of peptide-ssDNA complexes and consequently dictate the flow activation energy. In contrast, changing the chain length of either peptides or ssDNA did not alter the flow activation energy of the condensates but changed their viscoelastic properties. This is corroborated by coarse-grained molecular dynamics simulations, which revealed that while the peptide sequence alters the phase behavior, condensate density, and condensate viscosity, chain length variation does not alter the flow activation energy of the condensates. The observed changes in viscoelasticity upon chain length variation are likely due to the altered ssDNA chain relaxation dynamics in peptide-ssDNA condensates, as shown by our Rouse mode relaxation analysis (Fig. 5). Collectively, these results suggest that the quantification of the condensate flow activation energy through thermorheology can provide direct insights into the interchain interactions in the dense phase as well as the relative effects of chain length and valence in dictating the viscoelastic behavior of biomolecular condensates.

The simultaneous quantification of flow activation energy and viscoelasticity in our designed condensates allowed us to probe the distinct roles of intermolecular interactions and chain length on biomolecular transport in the dense phase. Unexpectedly, we observed that the translational mobility of peptide molecules within condensates does not scale with the bulk viscosity (Fig. 6D). Rather, diffusion measurements in the dense phase show that the peptide mobility depends on the flow activation energy of the condensate (Fig. 6D). This indicates that the translational motion of the peptides within condensates is reaction-limited rather than purely diffusive (*35*, *36*). These results highlight the utility of our combinatorial approach of measuring flow activation energy and viscosity in determining the mechanism of biomolecular transport within condensates. Our approach allows for a mechanistic explanation of the distinct dynamics of biomolecules within heterotypic biomolecular condensates that were reported previously (*83*, *84*). For example, Keenen *et al.* (*83*) studied the condensation of heterochromatin protein 1α (HP1α) with double-stranded DNA (dsDNA). Using FRAP experiments, the authors reported that HP1α exhibits identical dynamics within HP1α-dsDNA condensates irrespective of the size of the dsDNA (*83*). On the basis of our results reported here, we expect that increasing the dsDNA length enhances the viscosity of the condensates while leaving the flow activation energy of the condensates unchanged. The insensitivity of HP1α translational diffusion to the DNA chain length indicates that the protein diffusion within these condensates is primarily governed by a reaction-dominant mechanism. These observations, in conjunction with the results reported here, suggest that probing the diffusivity dynamics of macromolecules does not necessarily reflect the material properties of biomolecular condensates.

In a broader sense, heterotypic biomolecular condensates contain different types of macromolecules that come together through a complex interplay of chain-chain and chain-solvent interactions (*85*). Within a viscoelastic biomolecular condensate, macromolecules can exhibit distinct diffusivity dynamics depending on their interactions with the condensate-spanning viscoelastic network. As shown in this work and previous reports, the material properties of biomolecular condensates are sensitively dependent on several factors including sequence and chain length of component biomolecules, ionic strength, and pH among other factors (*15*, *22*–*24*, *73*, *79*). However, we observe that the diffusion of polypeptides within the dense phase can be insensitive to changes in condensate bulk material properties. On the basis of these results, we speculate that regulation of the macromolecule mobility rates within condensates can be achieved through a reaction-dominant transport mechanism or via assisted transport as observed in the case of nuclear pore complexes (*86*–*89*). In such cases, differential mobilities of macromolecules imparted by the viscoelastic network may provide a physical mechanism to tune functional outcome of biochemical processes in the condensate microenvironment while attenuating the undesired molecular mobilities.

In summary, our multiparametric approach encompassing optical tweezer–based microrheology, temperature-controlled fluorescence microscopy, and molecular dynamics simulations allowed us to shed light on the altered viscoelastic behavior of condensates upon sequence and length variation of constituent biopolymers. Our findings have implications on the way macromolecular diffusion within condensates is regulated. We envision that our experimental approach of quantifying condensate viscoelasticity along with flow activation energy will enable precise comparisons of biomolecular condensates that feature macromolecules with distinct sequences and sizes. Such understanding can ultimately enable rational strategies to engineer and manipulate the material and transport properties of biomolecular condensates through molecular designs.

## MATERIALS AND METHODS

The details of the materials used in this study as well as the protocols for sample preparation, passive pMOTs, temperature-controlled VPT, turbidity measurements, FRAP, and data analysis are provided in the Supplementary Materials.
