# Supplementary material for: Determinants of viscoelasticity and flow activation energy in biomolecular condensates
Source: Sci Adv. 2024 Feb 16;10(7):eadi6539. doi: 10.1126/sciadv.adi6539 (PMC10871536; doi:10.1126/sciadv.adi6539)
Supplement: Supplementary file 1 — Materials and Methods Figs. S1 to S15 Table S1 [file sciadv.adi6539_sm.pdf]

Science Advances
AAAS

Supplementary Materials for

Determinants of viscoelasticity and flow activation energy in biomolecular condensates

Ibraheem Alshareedah *et al.*

Corresponding author: Priya R. Banerjee, prbanerj@buffalo.edu; Ibraheem Alshareedah, ialshare@buffalo.edu; Davit A. Potoyan, potoyan@iastate.edu

Sci. Adv. **10**, eadi6539 (2024)
DOI: 10.1126/sciadv.adi6539

This PDF file includes:

Materials and Methods
Figs. S1 to S15
Table S1

Materials and Methods

Materials. ssDNA oligos of various lengths (20, 40, 90, and 200) were purchased from Integrated DNA Technologies (NJ, USA). The dry stocks were reconstituted in RNase-free water. The reconstituted solutions were centrifuged at 23000×g for 2 minutes to remove any particles and the supernatant was extracted. DNA concentration was measured subsequently using a NanoDrop 1C™ spectrophotometer. The DNA stocks were then aliquoted and stored at -20 ºC for further use. The peptides $[RPRPP]_5$, $[RGRGG]_5$, $[RGYGG]_5$, $[RGYGG]_3$, and $[RGYGG]_7$ were synthesized by Genscript inc, USA., and were reconstituted in RNase-free water containing 50 mM DTT (dithiothreitol, ThermoFisher Scientific). The purity of the peptides was higher than 90% as per manufacturer specifications. All the peptide sequences contain a C-terminal cysteine that is used for site-specific labeling with fluorescence dyes. Yellow-green carboxylate-functionalized polystyrene microspheres (diameter 0.2 and 1.0 μm) were purchased from Invitrogen. Cy5-labeled dT40 (IDT) was used for fluorescence imaging of the condensates.

Sample preparation. Peptide-ssDNA condensates were prepared by mixing appropriate amounts from concentrated stock solutions of the peptide and ssDNA to a final concentration of 5.0 mg/ml peptide and 5.0 mg/ml ssDNA. Thus, the mass ratio of the DNA to the peptide is 1:1 unless otherwise noted. This ratio was chosen based on turbidity measurements suggesting that the 1:1 ratio has maximal phase separation for all the systems tested (Fig. S1). The sample buffer contained 25 mM MOPS (pH 7.5), 25 mM NaCl, and 20 mM DTT. MOPS buffer was chosen because it has less susceptibility to pH change with temperature variations than the standard buffers such as Tris-HCl or HEPES (*91*). DTT was added to prevent cysteine oxidation. The condensate sample contained ~0.0004% (w/v) microspheres. We used 0.2 μm microspheres for the temperature-dependent particle tracking and 1.0 μm microspheres for the passive microrheology with optical tweezers (pMOT) measurements.

Passive microrheology with optical tweezers (pMOT). pMOT experiments were performed following our previously published protocol (*15*). Peptide-ssDNA condensate samples were placed on an 18 × 18 mm microscope coverslip and sandwiched with a 75 × 25 × 1 mm thick microscope glass slide using three layers of double-sided tape (Scotch 3M). Mineral oil was then injected into the chamber surrounding the sample from all directions to prevent sample evaporation with time. The sample was then loaded onto a correlative optical-tweezer and confocal microscopy setup (LUMICKS, C-Trap™). The sample was equilibrated for approximately 20 minutes until all droplets have settled on the coverslip surface and no fusion event was observed. By that time, several condensates were observed to contain 1-2 polystyrene beads. The optical trap was used to trap a bead within a condensate at minimal power (~10-50 μW). The trapped bead was then tracked using a brightfield camera at 500 Hz using a template-matching algorithm for 10-30 minutes. Approximately 20 condensates over three independent sample preparations were tested. Further details of pMOT experiments, including various control measurements, are reported in our earlier work (*15*).

Temperature-controlled video particle tracking (VPT). Peptide-ssDNA condensate samples were sandwiched between a coverslip and a glass slide similar to what is described in the pMOT section. The sandwich was then placed on a custom-built thermal stage (INSTEC) which is

attached to a Zeiss primovert inverted microscope with a 100x oil-immersion objective lens. Teledyne FLIR blackfly S USB3 CMOS camera was used for imaging. Condensates were allowed to settle on the glass slide for approximately 20 minutes prior to the start of the measurement. Each condensate contained about 20-50 fluorescent microspheres (200 nm). The temperature stage was first set to the lowest experimental temperature (5 ºC). Due to the objective acting as a heat sink, the actual temperature within the sample was different from the preset temperature of the thermal stage. To obtain an accurate measurement of the sample temperature, we used a thermocouple with a heat insert that touches the glass directly inside the thermal stage chamber on the edge of the condensate sample. Within 10-20 minutes, the temperature reading of the thermocouple was observed to be equilibrating around 10 ºC (preset temperature = 5 ºC). To start imaging, the microscope objective was focused on a particular condensate. A time-lapse video was collected for 2000 frames at a rate of 10 frames per second (exposure time is set to 100 ms). For condensates that showed high viscosity and slow motion of the particles, a frame time of 200 ms was used. The temperature was increased in steps of 5 ºC and the sample was left to equilibrate for 10 minutes at each temperature. A similar time-lapse video of the same condensate was collected to record the motion of the particles at the new temperature. This procedure was repeated to collect 5-7 points between 11 ºC and 70 ºC (preset temperature = 90 ºC). Three trials were done for three independent sample preparations.

Turbidity measurements. Samples were prepared in a tube by mixing the peptide and the ssDNA at the desired mixing ratio and a fixed peptide concentration of 1.0 mg/ml. The buffer of these samples contains 25 mM MOPS (pH 7.5), 25 mM NaCl, and 20 mM DTT. The sample was then placed on a UV-Vis spectrophotometer (NanoDrop 1C) and the solution turbidity at 350 nm was measured for three independent samples. Before measuring the turbidity of peptide-ssDNA samples, the instrument was blanked using the experimental buffer. The values of the turbidity were averaged for each mixing ratio and the error was estimated as half the range of experimentally measured values.

Fluorescence Recovery after Photobleaching (FRAP). Samples were prepared at 5.0 mg/ml concentration of both peptide and ssDNA in a buffer containing 25 mM MOPS (pH 7.5), 25 mM NaCl, and 20 mM DTT. To measure peptide diffusion within $[RGRGG]_5$-ssDNA condensates, we added ~400 nM of the Alexa488-labeled $[RGRGG]_5$ peptides to the buffer before mixing the other components. Similarly, we used Alexa488-labeled $[RGYGG]_5$ peptides for $[RGYGG]_5$-ssDNA condensates. 500 nM of the Cy5-labeled dT40 was used to measure the diffusion within $[RGYGG]n$-dT40 condensates for $n = 3$, 5, and 7 (Fig. S15). The final sample was then sandwiched between a glass slide and a coverslip using double-sided tape. Mineral oil was inserted into the chamber to surround the sample and prevent evaporation. The sample chamber was then placed on a confocal microscope (LUMICKS, C-Trap™). For all FRAP experiments, we fixed the bleaching region to 0.6 × 0.6 μm. This was done to ensure that the changes in the FRAP recovery time are only due to the probe dynamics and not due to the larger bleaching area (*67, 70, 71*). Further, we performed the FRAP experiments only on condensates with diameters that are substantially larger than 3 μm (at least 5× bleaching ROI radius) to rule out interfacial resistance effects (*67*). For each sample, we measured the FRAP traces for 4-6 condensates. The recovery traces were corrected for photofading and normalized with respect to the bleaching depth following

previously published procedures (*72*). The recovery traces were then averaged, and the error was estimated as the standard deviation at each time point. To estimate the recovery half-time, we fitted individual recovery traces using the following equation (*70, 72*)

$$I_{recovery}(t) = \frac{I_0 + I_\infty \frac{t}{\tau_{1/2}}}{1 + \frac{t}{\tau_{\frac{1}{2}}}} \tag{1}$$

Here, I_0, I_∞, and $\tau_{1/2}$ are fitting parameters, the latter represents the FRAP recovery half-time. The diffusion timescale was then calculated by dividing the recovery half-time by the square of the radius of the bleaching area ($\tau_D = {\tau_{\frac{1}{2}}}/{R^2}$). The reported FRAP diffusion timescale was taken as the average of the values extracted from the fits of individual FRAP measurements. The error was estimated as the standard deviation of the values.

Data analysis: pMOT. The output of the pMOT experiments is two-dimensional trajectories of trapped particles within condensates. These trajectories are analyzed to calculate the complex modulus of the condensate as a function of frequency ω

$$G^*(\omega) = G'(\omega) + i\, G''(\omega) \tag{2}$$

Where G' and G'' are the frequency-dependent elastic and viscous moduli, respectively. Briefly, the trajectories are first detrended using a spline-based detrending algorithm to remove the long-time drift of the trajectories. Next, each component (X and Y) of the trajectory is treated separately. For each one-dimensional trajectory (X or Y), we obtain the trap stiffness using the equipartition theorem (*44, 92, 93*)

$$\kappa_x = k_B T / \langle x^2 \rangle \tag{3}$$

Where κ_x is the optical trap stiffness in the x direction, T is the temperature in Kelvin, and k_B is the Boltzmann constant. Next, a normalized position autocorrelation function $g(\tau)$ is calculated. The autocorrelation function value at $\tau = 0$ is extrapolated using a spline algorithm. The autocorrelation function is then transformed into the frequency domain $\hat{g}(\omega)$ using a discrete Fourier transform algorithm (*44, 92, 93*)

$$-\omega^2 \hat{g}(\omega) = i\omega g(0) + \frac{\left(1 - e^{-i\omega t_1}\right)\left(g_1 - g(0)\right)}{t_1} + \dot{g}(\infty) e^{-i\omega t_N} + \sum_{k=2}^{N} \left(\frac{g_k - g_{k-1}}{t_k - t_{k-1}}\right)\left(e^{-i\omega t_{k-1}} - e^{-i\omega t_k}\right) \tag{4}$$

Lastly, the complex modulus is calculated using (*44, 92, 93*)

$$G^*(\omega) = G'(\omega) + i\, G''(\omega) = \frac{\kappa}{6\pi a} \left(\frac{i\omega \hat{g}(\omega)}{1 - i\omega \hat{g}(\omega)}\right) \tag{5}$$

Where a is the radius of the particle (0.5 μm). For each trial, we extract two sets of G' and G'' values for each frequency. The total number of G' and G'' sets is ~40 sets for ~20 condensates over three independent sample preparations. Finally, at each frequency, we average the values of G' and G'' and report the final value. The error is calculated as the standard deviation. For all the systems tested, we exclude the top and bottom 5% of the values to eliminate any outliers. Further details of the experiment and data analysis, including various controls for the effects of condensate surface and glass surface on the measurement, are reported in our earlier work (*15, 92*).

Data analysis: Temperature-controlled video particle tracking. Movies of condensates containing diffusing polystyrene particles at different temperatures were processed first using Fiji-ImageJ software (*94*). Each movie contained about 2000 frames. The particles were tracked using the TrackMate software plug-in in Fiji (*95*). In particle tracking, intensity filters were used to exclude any particle aggregates from tracking. The extracted trajectories were corrected for drifting by subtracting the center of mass trajectory, which was calculated using the velocities of particles in the following way

$$\boldsymbol{X_{COM}}(k) = \boldsymbol{X_0} + \sum_{j=0}^{k} \frac{1}{N_j} \sum_{i=1}^{N} \boldsymbol{v_{i,j}} \quad (6)$$

Where k is the frame number at which $\boldsymbol{X_{COM}}$ is calculated, j is the individual frame, and N_j is the number of particles in frame j. $\boldsymbol{X_0}$ is the center of the mass vector of the first frame. After correcting the trajectories for drifting, the mean squared displacement (MSD) was calculated as

$$MSD(\tau) = \langle \boldsymbol{R}(t+\tau) - \boldsymbol{R}(t) \rangle_{t,N} \quad (7)$$

Where τ is the lag time and $\boldsymbol{R}$ is the position vector of the particle. The MSD was then fitted using (*15, 96*)

$$MSD(\tau) = 4D\tau^{\alpha} + N \quad (8)$$

to obtain the diffusion coefficient D of the particles. For all the systems, we ensured that the value of the diffusivity exponent α is equal to 1 by choosing an appropriately long frame time to ensure measuring the terminal viscous behavior. The diffusion coefficient is then converted to viscosity using the Stokes-Einstein equation (*15, 96*)

$$\eta = \frac{k_B T}{6\pi D R} \quad (9)$$

where R is the radius of the particles and T is the temperature of the sample. This analysis was done for movies collected at different temperatures and the value of the viscosity was plotted against the temperature. To obtain the activation energy, we plotted $\ln \eta$ against $1/T$ and fitted the resulting data to Equation 2 in the main text. Each peptide-ssDNA condensate system was tested three times in three different sample preparation. The average value of the activation energy is reported, and the error is estimated as half the range of values. As a control, we measured the viscosity of the Water-Glycerol solution at 90% glycerol using our VPT-based approach and

compared it with the published values in the literature (*51, 52*), which revealed good agreement (see Fig. 6).

Atomistic simulation

Umbrella sampling simulations: The structure of a single-stranded poly thymine DNA chain containing 8 residues (dT8) was generated using X3DNA. Pentapeptides of RGYGG, RGRGG, and RPRPP were generated using PeptideBuilder. We placed one dT8 chain and one peptide chain in a cubic box of 7 nm. After that, 100 mM of ions (Na^+ or Cl^-) and water are added to fill the square box. After preparing a solvated box containing protein, ssDNA, and ions, the system was subjected to energy minimization with the conjugate-gradient algorithm via OpenMM 7.6 library using a99SBdisp-ILDN and TIP3P water force fields. Following energy minimization steps, we have run 4 ns NVT and 4 ns NPT equilibration runs at temperature T = 300 K. In all these simulations, we applied a leapfrog integrator with a time step of 2 fs and a Nose–Hoover thermostat to maintain an average temperature with a relaxation time of 0.5 ps. We used the Parrinello-Rahman barostat to maintain an average pressure of 1 bar with a coupling time constant of 0.5 ps for NPT equilibration. All simulations used periodic boundary conditions in all directions, and the neighboring list was modified every 10 steps using a grid system with a 1 nm short range. The LINCS algorithm was used to restrict all bonds.

To obtain free energy profiles of unbinding we have carried out umbrella sampling starting from equilibrated configurations of ssDNA-peptide systems by using the center of mass distance between ssDNA and peptide chains. A force constant of 600 kJ/mol/nm^2 was applied. We applied harmonic bias at distances ranging from 0.4 Å to 3.4 Å with an interval of 0.085 Å (in total 35 windows). Each window has been simulated for 40 ns.

Simulated tempering simulation: To computationally mimic the condensate environment we next prepared multi-chain simulations using box size of 12 nm, 4 nm, and 4 nm, each containing dT8 and RGYGG, dT8 and RGRGG, and dT8 and RPRPP, at a density of 45 mg/ml with help of Packmol. After that, 100 mM of ions (Na^+ or Cl^-) and water are added to fill the box. After preparing a solvated box containing peptide, dT8, and ions, the system was subjected to energy minimization with the conjugate-gradient algorithm via OpenMM 7.6 library using a99SBdisp-ILDN and TIP3P water force fields. Following energy minimization steps, we have run 4 ns NVT simulation followed by 4 ns NPT simulations at temperature T = 300 K. In all these simulations, we applied a leapfrog integrator with a time step of 2 fs and a Nose–Hoover thermostat to maintain an average temperature with a relaxation time of 0.5 ps. We used the Parrinello-Rahman barostat to maintain an average pressure of 1 bar with a coupling time constant of 0.5 ps for NPT equilibration. All simulations used periodic boundary conditions in all directions, and the neighboring list was modified every 10 steps using a grid system with a 1 nm short range. The LINCS algorithm was used to restrict all bonds. We run 25 replicas at the temperature range of 300 K to 500 K. Total production run simulation was performed over 2 microseconds, at an exchange of 2000 steps. We used MDAnalysis library for extracting peptide-DNA contacts and radial distribution functions.

Coarse-grained molecular dynamics simulation

Direct coexistence simulation: We used the hydrophobicity scale model (HPS) (*97*) which coarse-grains each residue and nucleobase as single beads and decomposes pairwise interaction between beads into short-range hydropathy contact modeled by Ashbaugh-hatch potential and long-range electrostatic forces modeled by Debye Huckel potential. We have used the recent parameterization of hydrophobicity scales named CALVADOS2 (*63*) and extended it by introducing dT beads with a hydrophobicity scale of 0.01. Simulations have used 0.2 nm cutoff for all short-range interactions and 0.4 nm cutoff for all long-range interactions (*15*).

To map phase-diagrams of condensates we have carried out direct coexistence simulations over a range of temperatures. Box size was set to L_x=L_y=15 nm and L_z=150 nm. We have constructed all mixtures by packing 400 peptides with appropriate number of nucleotides by keeping the polyelectrolyte charge ratio fixed at q_+/q_-=1.25. Simulations were done using Langevin integrator with 0.01 ps time step. Simulations were run for over 5 x10^7 steps for each temperature. First 10^6 steps are discarded as equilibration with the rest of the trajectory used for measuring the density of dilute and dense phases. We determined the critical temperature *Tc* by fitting the density profiles to the critical temperature profile of 3D Ising model and used the method of rectilinear diameters to find the critical density.

Viscosity calculation via equilibrium NVT simulations: To calculate viscosities of condensates we have carried out simulations by generating condensates in cubic boxes using the biomolecular densities extracted from the direct coexistence simulations. We used box size of L_x=L_y=L_z=25 nm to carry out NVT simulations with Langevin integrator using 0.01 ps time step for 10^7 steps. The pressure tensor was logged every 0.1 ps time interval. The viscosity was calculated using pressure tensor autocorrelation function to compute relaxation modulus *G(t)* according to Green-Kubo relations (*64*). We have then carried out direct integration of *G(t)* to compute viscosity. We have also quantified condensate viscosity by computing relaxation modulus *G(t)* via the Rouse mode autocorrelation function of ssDNA chains (*64*) using the same simulation data.

The stress tensor *G(t)* can be determined by computing the autocorrelation of any of the off-diagonal components ($\alpha\beta$) of the pressure tensor at equilibrium:

$$G(t) = \frac{V}{k_B T}\langle \sigma_{\alpha\beta}(t)\sigma_{\alpha\beta}(0)\rangle$$

Our system is isotropic; hence a more accurate expression of *G(t)* can be obtained by using the six independent components of the pressure tensor:

$$G(t) = \frac{V}{5k_B T}\langle \sigma_{xy}(t)\sigma_{xy}(0) + \sigma_{yz}(t)\sigma_{yz}(0) + \sigma_{xz}(t)\sigma_{xz}(0) + \frac{1}{6}\left[\delta_{xy}(t)\delta_{xy}(0) + \delta_{yz}(t)\delta_{yz}(0) + \delta_{xz}(t)\delta_{xz}(0)\right]\rangle,$$ where $\delta_{\alpha\beta} = \sigma_{\alpha\alpha} - \sigma_{\beta\beta}$.

The mechanical stress can also be calculated from the Rouse modes of a single chain:

$$\sigma_{\alpha\beta}(t) = \frac{\rho k_B T}{N} \sum_{p=1}^{N-1} \frac{\langle X_{p\alpha}(t) X_{p\beta}(t) \rangle}{\langle X_{p\alpha}^2 \rangle_{eq}}$$

Where the *p*-th Rouse mode is $X_p(t) = \frac{1}{N} \sum_{n=1}^{N} r_n(t) \, cos\left(\left(n - \frac{1}{2}\right) p\pi/N\right)$, *p*=0, 1, …, *N*-1; with *N* being the chain length, ρ the monomer number density, and r_n the position of the *n*-th monomer.

Supplementary Tables

System	E_A (RT)	E_A (RT)	E_A (RT)	$E_A \pm \Delta E_A$ (RT)
$[RPRPP]_5$-dT40	10.35	8.44	8.72	9 ± 1
$[RGRGG]_5$-dT40	15.97	18.87	17.23	17 ± 1
$[RGYGG]_3$-dT40	27.01	22.07	21.58	24 ± 3
$[RGYGG]_5$-dT40	23.27	29.05	25.96	26 ± 3
$[RGYGG]_7$-dT40	27.98	23.03	23.38	25 ± 2
$[RGRGG]_5$-dT20	19.69	18.44	19.00	19.0 ± 0.6
$[RGRGG]_5$-dT90	18.44	17.78	18.48	18.2 ± 0.4
$[RGRGG]_5$-dT200	19.12	19.18	17.55	18.6 ± 0.8

Table S1. Activation energy values for all the peptide-ssDNA condensates tested in this study. Each sample was measured three times with independent sample preparations. The reported value is the average of the three measurements and the error is calculated as half the range of values. The units are RT at T = 25 ºC, where R is the universal gas constant (1 RT is 2.479 kJ/mol).

Supplementary Figures

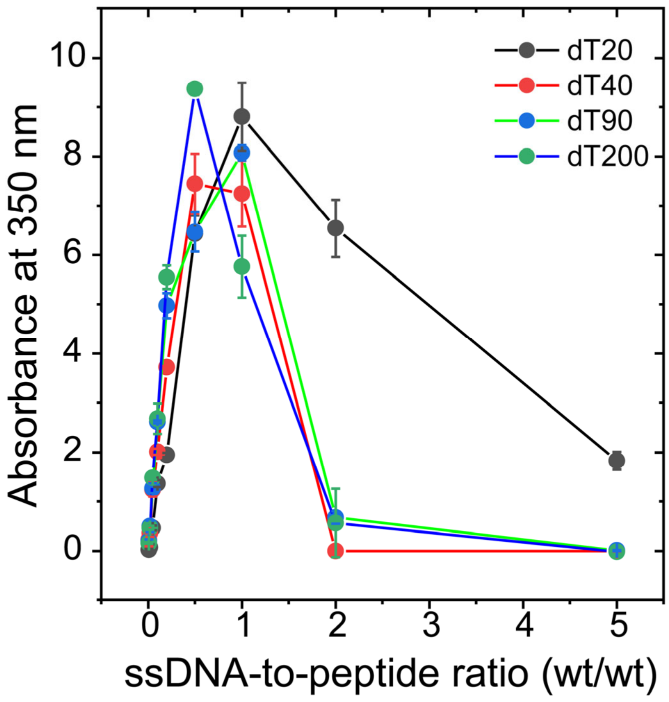

Figure S1. A plot showing absorbance at 350 nm (turbidity) of [RGRGG]$_5$-dTn mixtures at different mixing stoichiometries. dTn is a poly(dT) ssDNA with length n equals 20, 40, 90, or 200.

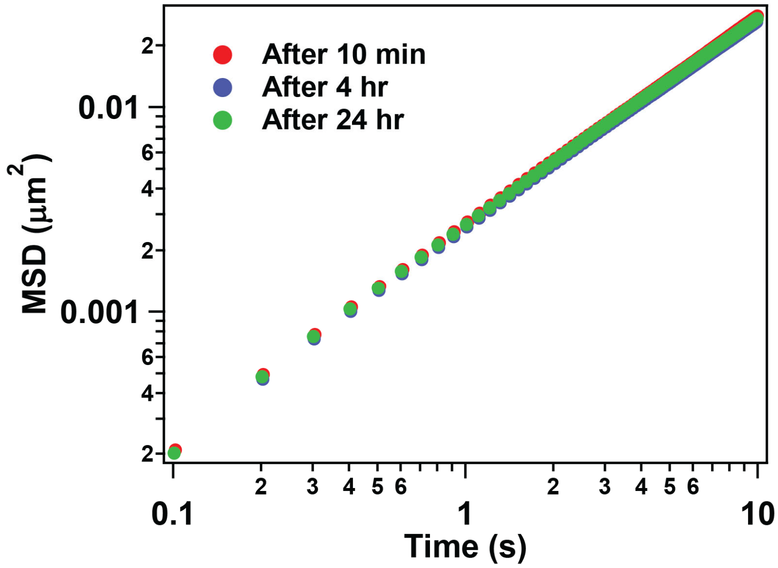

Figure S2. **Peptide-ssDNA condensates exhibit Maxwell fluid-like behavior without any sign of physical aging.** Ensemble averaged MSDs of 200 nm particles within [RGRGG]$_5$-dT40 condensates at 25 °C after 10 min, 4 hrs, and 24 hrs of sample preparation. The identical ensemble average MSDs at all ages reveal that these condensates do not physically age during this time period.

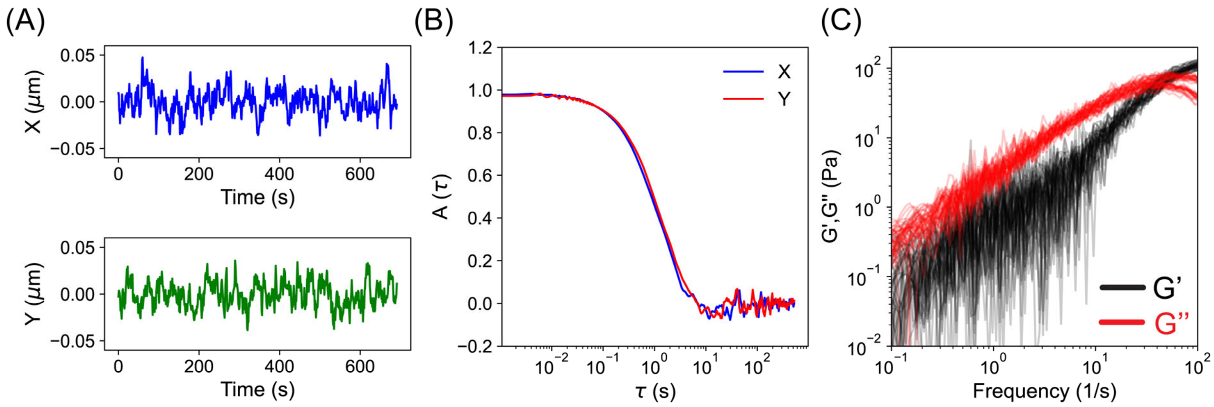

Figure S3. Passive microrheology with optical tweezer (pMOT) of condensates. **(A)** Representative trajectories of a trapped bead within a [RGRGG]5-dT40 condensate in the X and Y directions. **(B)** Position autocorrelation curves of the bead motion in the X and Y directions. **(C)** Viscoelastic moduli for twenty [RGRGG]5-dT40 condensates. The reported data in Figure 1B in the main text is the average of these moduli.

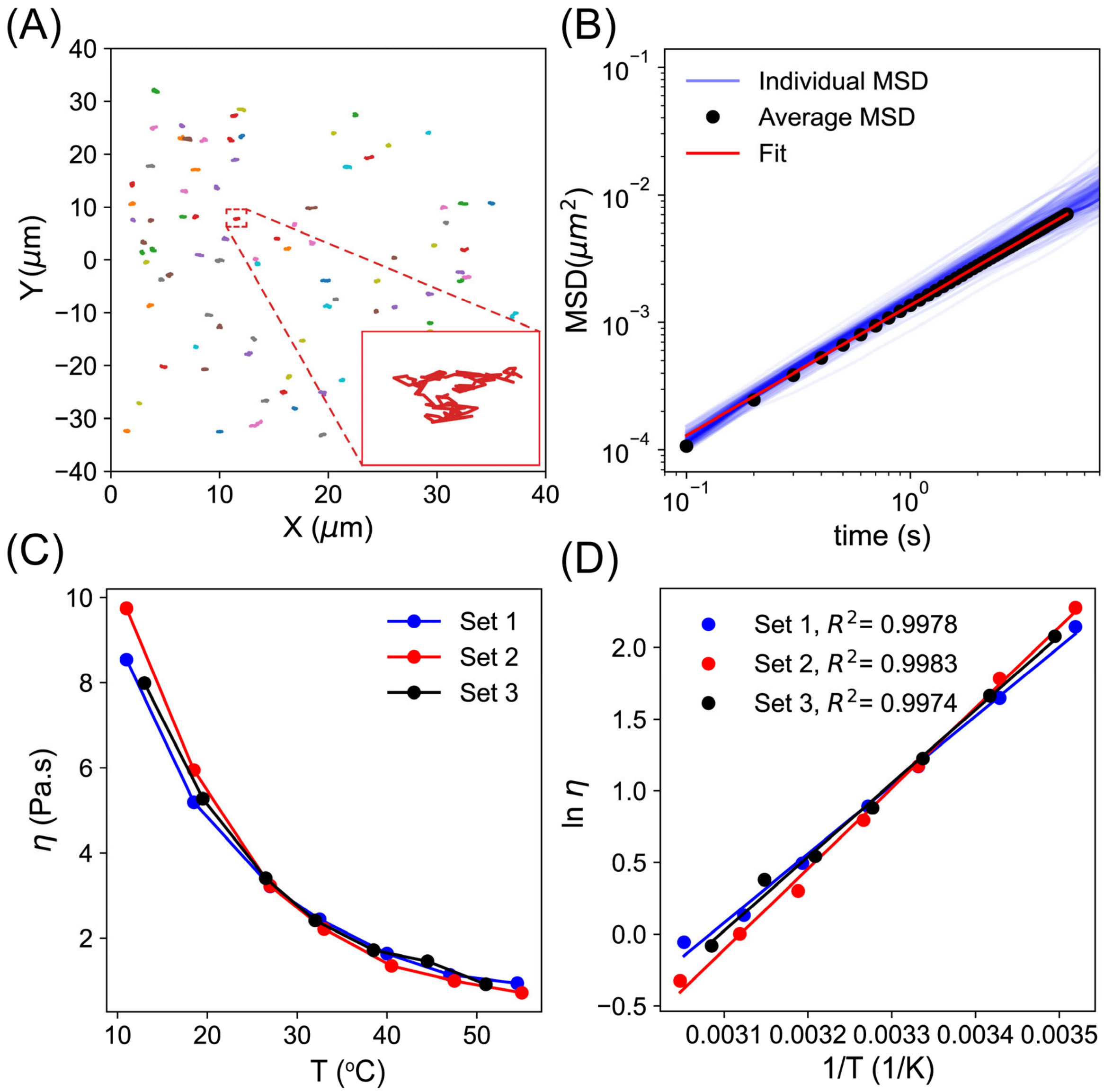

Figure S4. Temperature-controlled VPT-based determination of flow activation energy of peptide-ssDNA condensate. **(A)** Representative trajectories of 200 nm beads within a [RGRGG]$_5$-dT40 condensate as measured by video particle tracking at room temperature. **(B)** Individual MSD plots for all the beads within the condensate shown in (A). The black symbols are the ensemble-averaged MSD. The red line is a fit of the data according to Equation 8. **(C)** Viscosity variation with temperature for three independently prepared [RGRGG]$_5$-dT40 condensates. **(D)** Arrhenius plots for the data shown in (C) for the three trials. The lines are fits to the data using Equation 2 in the main text. The corresponding activation energy values are reported in Supplementary Table S1.

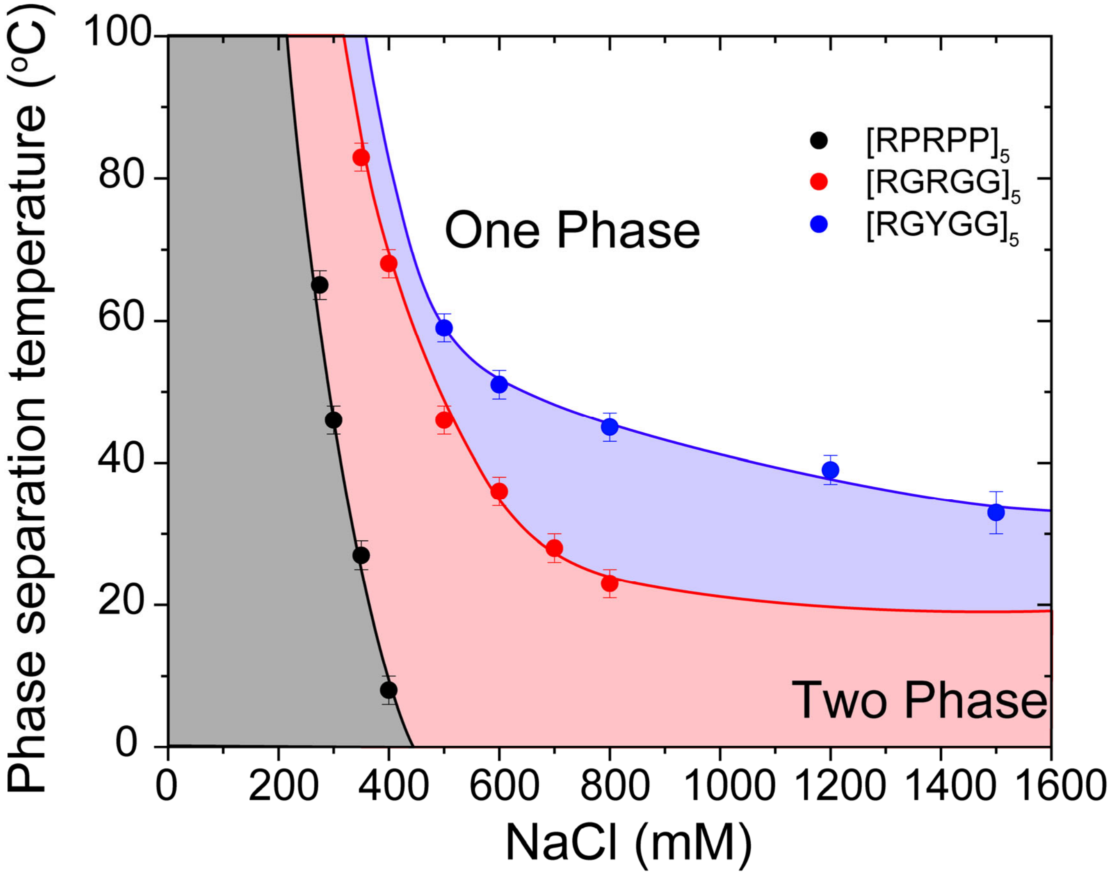

Figure S5. Temperature−salt phase diagrams of peptide-rU40 condensates. This data is repurposed from our previous work (*15*). The shaded regions indicate temperature and salt conditions where phase separation occurs. All samples were prepared at 5 mg/ml polypeptide and 2.5 mg/ml rU40 RNA (analogous to dT40) in 25 mM MOPS, 25 mM NaCl, and 20 mM DTT. Note that both $[RGRGG]_5$ and $[RPRPP]_5$ have similar stickers (Arg residues) even though they exhibit vastly different material properties as well as upper critical solution temperatures (*15*) (Fig. 2 in the main text). This can be attributed to the fact that spacers modulate peptide interactions with nucleic acids through the effective solvation volume and/or steric forces associated with the peptide-ssDNA complexes. Further, our observations that the flow activation energy decrease when mutating Gly spacers to Pro spacers confirm that spacers affect the intermolecular interactions between the peptide and the ssDNA chain as well as the chain solvation properties. Accordingly, there are further nuances to the sticker-spacer classification of residues beyond what is considered in this work.

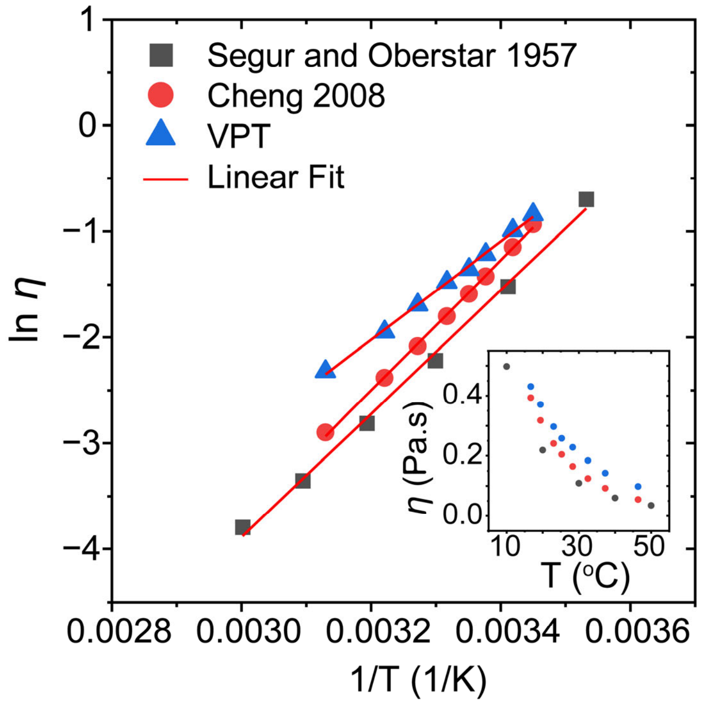

Figure S6. A comparison between temperature-dependent viscosities of Glycerol-Water mixtures measured in this study *vs.* reported in previous studies. Arrhenius plots for the viscosity of Glycerol-Water mixtures as obtained from our VPT measurements (blue triangles; this study), a previous study by Segur and Oberstar (*51*) (black squares), and the viscosity formula from Cheng (*52*). Red lines are linear fits according to Equation 2 in the main text. The obtained values of the activation energy are 15.6, 20.7, and 19.6 RT (38.7, 51.2, and 48.7 kJ/mol), respectively. The inset shows the viscosity value as a function of temperature for the same data sets.

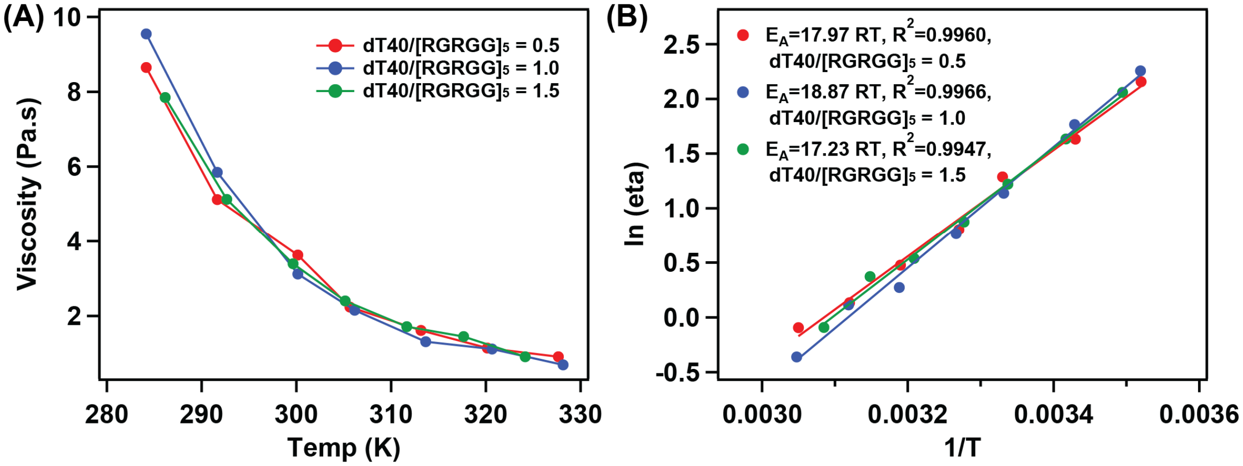

Figure S7. Flow activation energy measurements for condensates prepared at various ssDNA to peptide mass ratios. (**A**) Viscosity variation with temperature for $[RGRGG]_5$-dT40 condensates prepared at three different mass ratios of dT40/$[RGRGG]_5$ = 0.5, 1.0, and 1.5. **(B)** Arrhenius plots for these $[RGRGG]_5$-dT40 condensates. The solid lines represent the fits to the plots from which the activation energy for the condensates prepared at different mass ratios of $[RGRGG]_5$ and dT40 were determined.

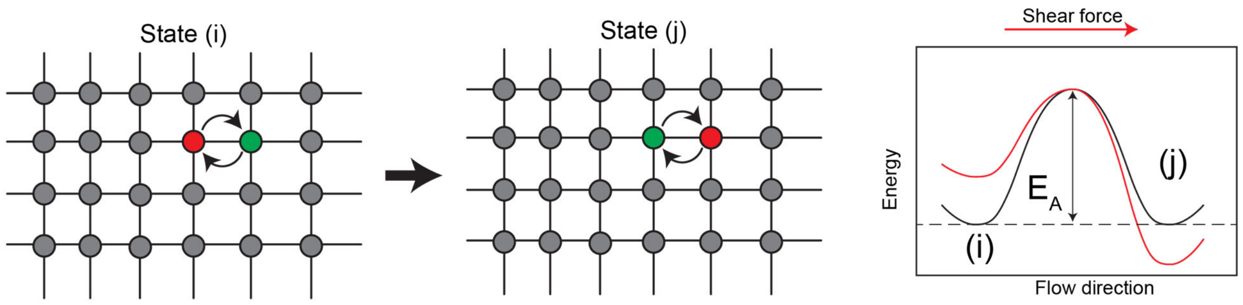

Figure S8. A schematic showing the activation energy as the energy barrier for molecular exchange within a fluid. This barrier is associated with the energy needed to reconfigure the molecules from state (i) to state (j). When a shear force is applied from left to right (red arrow), the barrier becomes asymmetric with the state (j) having lower energy, and hence directional diffusion/flow is favored.

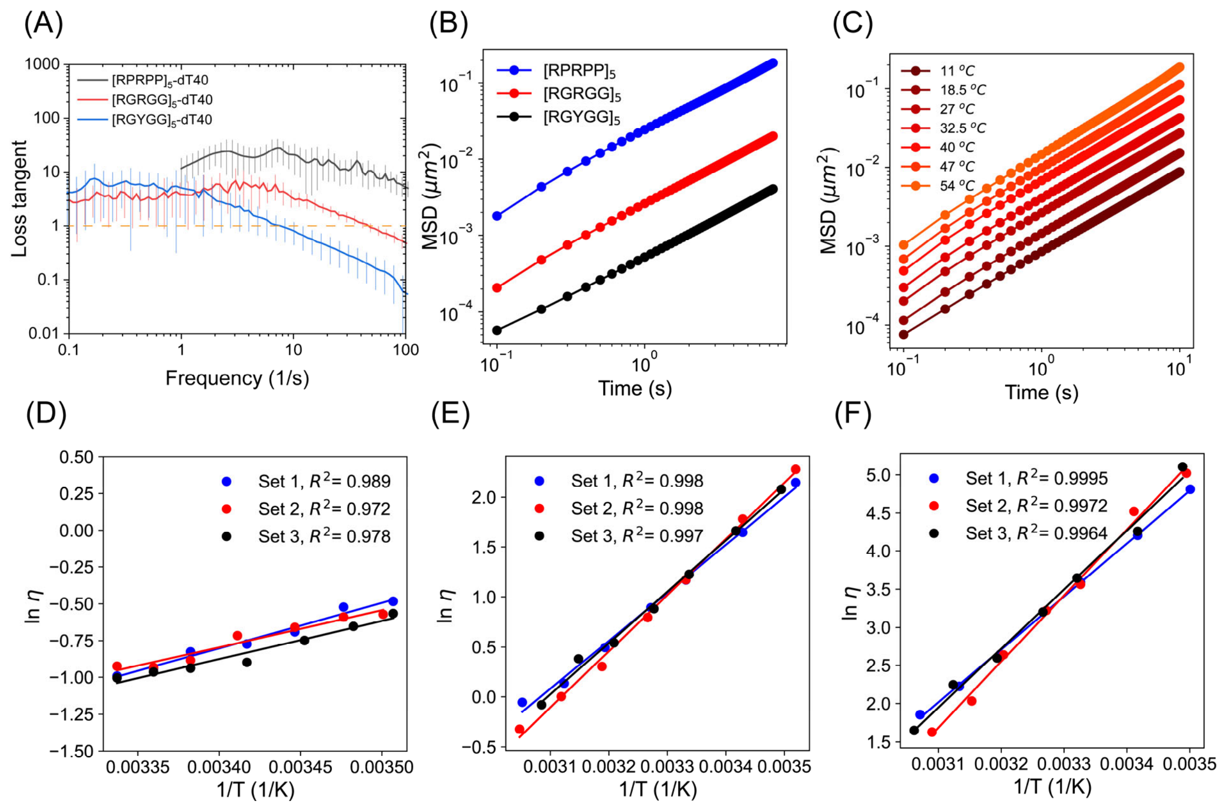

Figure S9. Viscoelasticity and activation energy of peptide-dT40 condensates. **(A)** Loss tangent (G''/G') of peptide-ssDNA condensates showing distinct terminal relaxation times depending on the peptide sequence. When the loss tangent is less than 1, the elastic modulus dominates the rheological response. We observe that the loss tangent for [RGYGG]$_5$-dT40 system crosses 1 at a frequency of ~10 Hz, indicating a terminal relaxation time of ~100 ms. For [RGRGG]$_5$-dT40, the terminal relaxation time is ~ 20 ms. Lastly, the [RPRPP]$_5$-dT40 condensates have a loss tangent that is greater than 1 over the entire range of experimental frequencies, indicating a dominant viscous response. **(B)** Ensemble-averaged MSD of 200 nm particles within peptide-dT40 condensates. The peptides shown here are [RGRGG]$_5$, [RPRPP]$_5$, and [RGYGG]$_5$. **(C)** Ensemble-averaged MSD of 200 nm particles within a [RGRGG]$_5$-dT40 condensate at different temperatures. **(D, E, F)** Arrhenius plots for three independently prepared peptide-dT40 condensates for the three peptides; [RPRPP]$_5$, [RGRGG]$_5$, and [RGYGG]$_5$, respectively. The lines are fits to the data using Equation 2 in the main text. The corresponding activation energy values are reported in Supplementary Table S1.

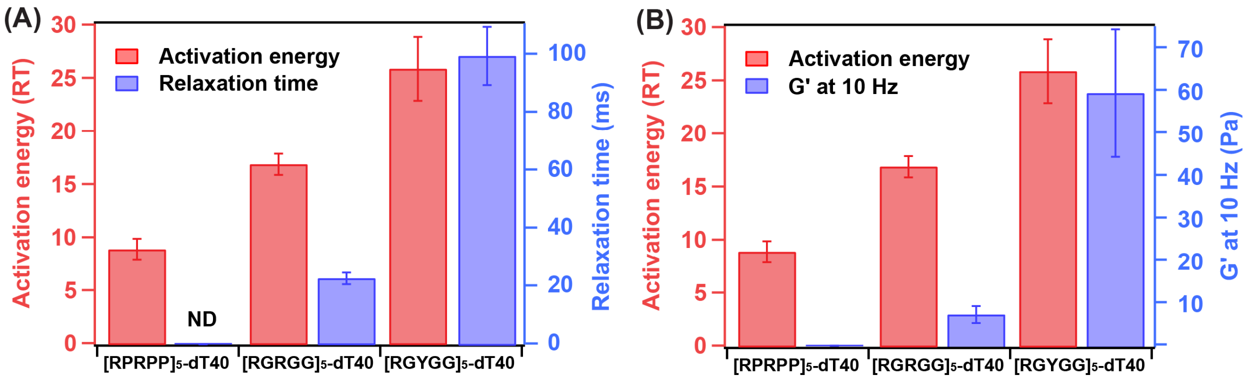

Figure S10. Effect of peptide sequence on the activation energy and frequency-dependent viscoelasticity of condensates. (A) A comparison between activation energy and the terminal relaxation time obtained from the G', G'' crossover frequency. ND in the figure means that the crossover frequency for $[RPRPP]_5$-dT40 condensates is > 100 Hz and hence could not be determined within the accessible frequency range of our microrheology measurements. **(B)** A comparison between the activation energy and the elastic modulus (G') at 10 Hz.

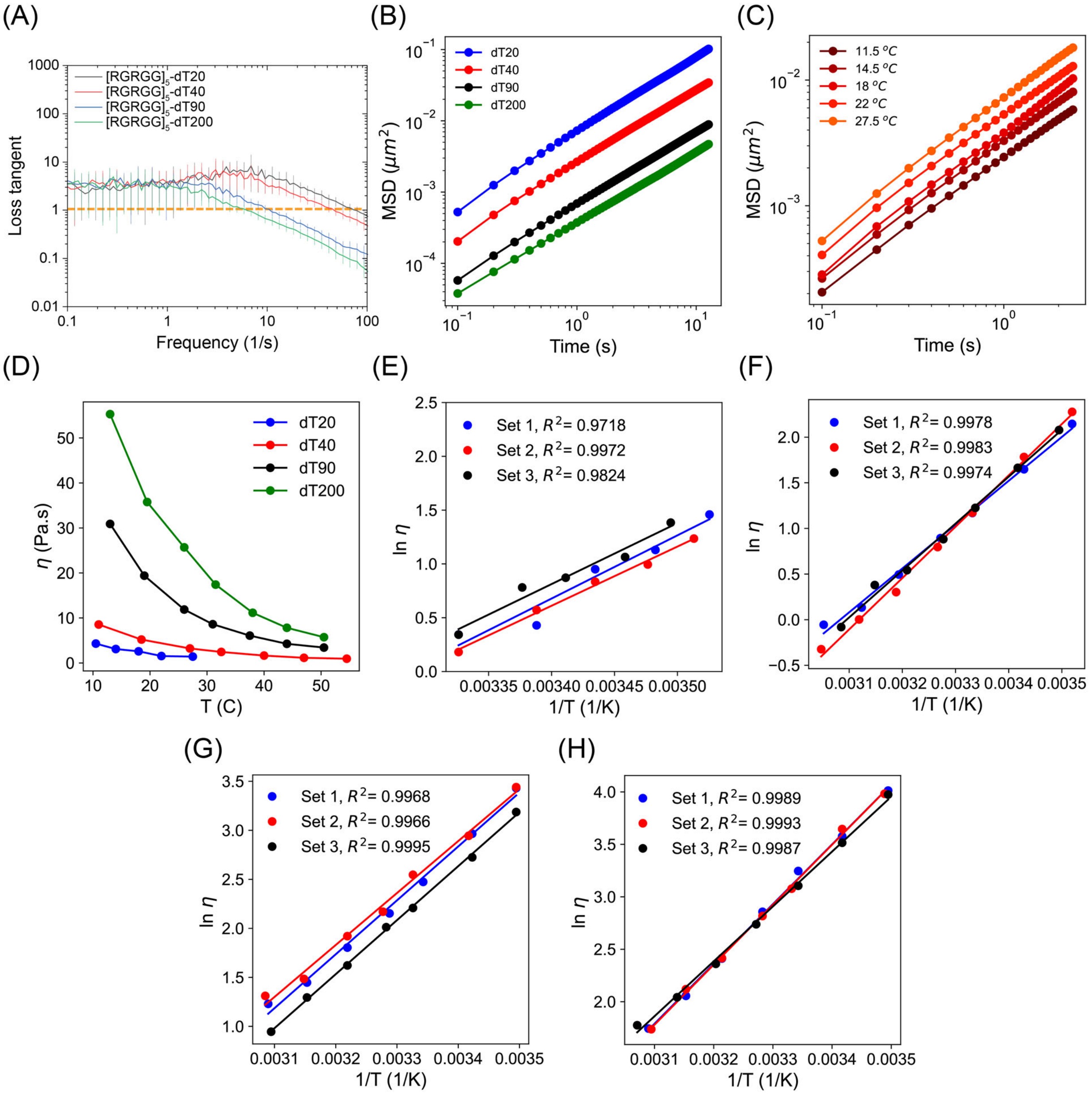

Figure S11. Viscoelasticity and activation energy of [RGRGG]5-ssDNA condensates. (A) Loss tangent (G''/G') of four peptide-DNA condensates in (A-D) showing distinct terminal relaxation times with DNA length variation. **(B)** Ensemble averaged MSD of 200 nm particles within [RGRGG]5-dTn condensates at 27 °C where n is the length of the ssDNA. Data are shown for n = 20, 40, 90, and 200. **(C)** Ensemble-averaged MSD of 200 nm particles within [RGRGG]5-dT20 condensates at different temperatures. **(D)** Viscosity variation with temperature for [RGRGG]5-dTn condensates, where n is the length of the ssDNA. The DNA lengths tested are 20, 40, 90, and 200. These are the same data as shown in Fig. 4a in the main text. **(E-H)** Arrhenius plots for three independently prepared [RGRGG]5-dTn condensates where $n = 20$, 40, 90, and 200, respectively. The lines are fits to the data using Equation 2 in the main text. The corresponding activation energy values are reported in Supplementary Table S1.

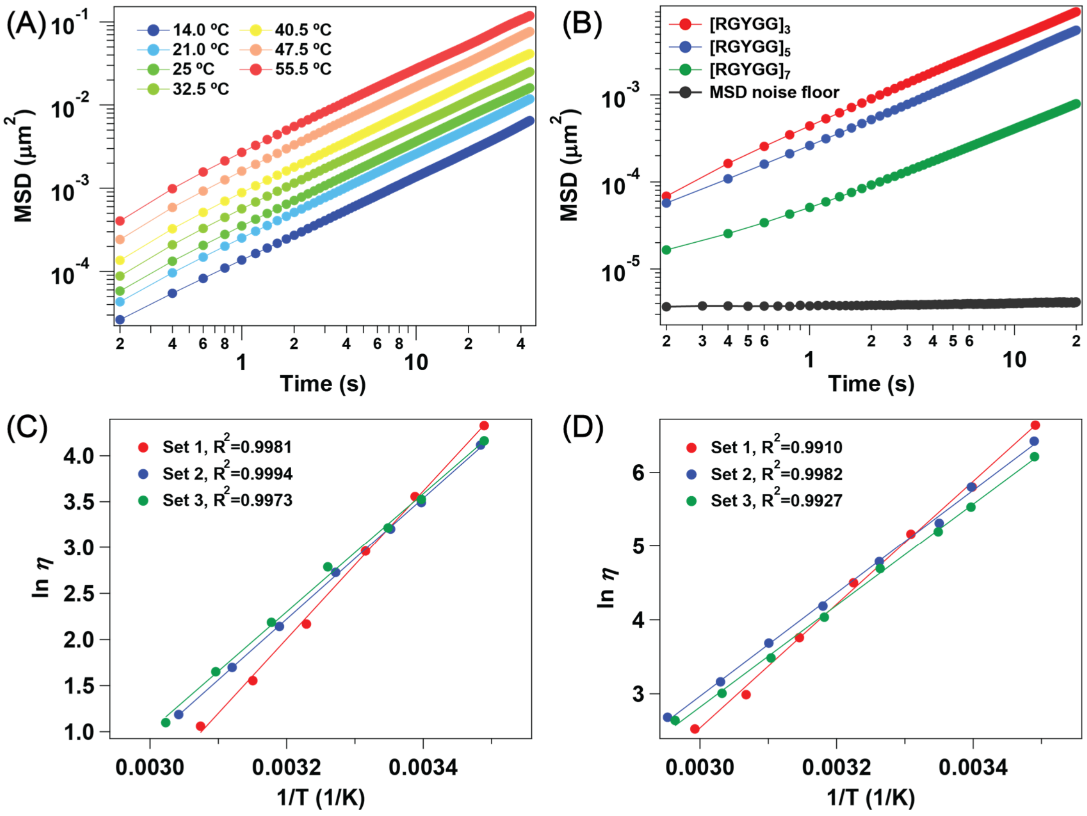

Figure S12. Effect of peptide repeat length on the activation energy of condensates. (A) Ensemble-averaged MSD of 200 nm particles within $[RGYGG]_3$-dT40 condensates at different temperatures. **(B)** Ensemble averaged MSD of 200 nm particles within $[RGYGG]n$-dT40 condensates at 27 °C where n is the repeat length of the [RGYGG] peptide. Data are shown for n = 3, 5, 7, and for the noise floor of the MSDs. **(C-D)** Arrhenius plots for three independently prepared $[RGYGG]n$-dT40 condensates where n = 3 and 7, respectively. The lines are fits to the data using Equation 2 in the main text. The corresponding activation energy values are reported in Supplementary Table S1.

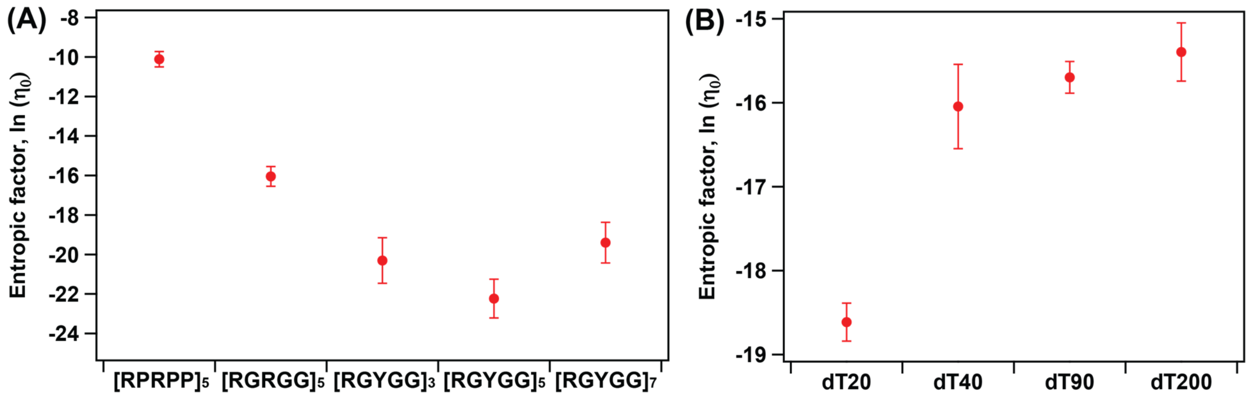

Figure S13. The pre-exponential factors of different peptide-ssDNA condensates. The extrapolated entropic factor of (**A**) peptide-dT40 condensates from the data shown in Fig. 2E and Fig. 4B in the main text; (**B**) $[RGRGG]_5$-dT*n* condensates from the data shown in Fig. 3G in the main text. The entropic factors are calculated from the intercept of the linear fit according to Equation 2 in the main text.

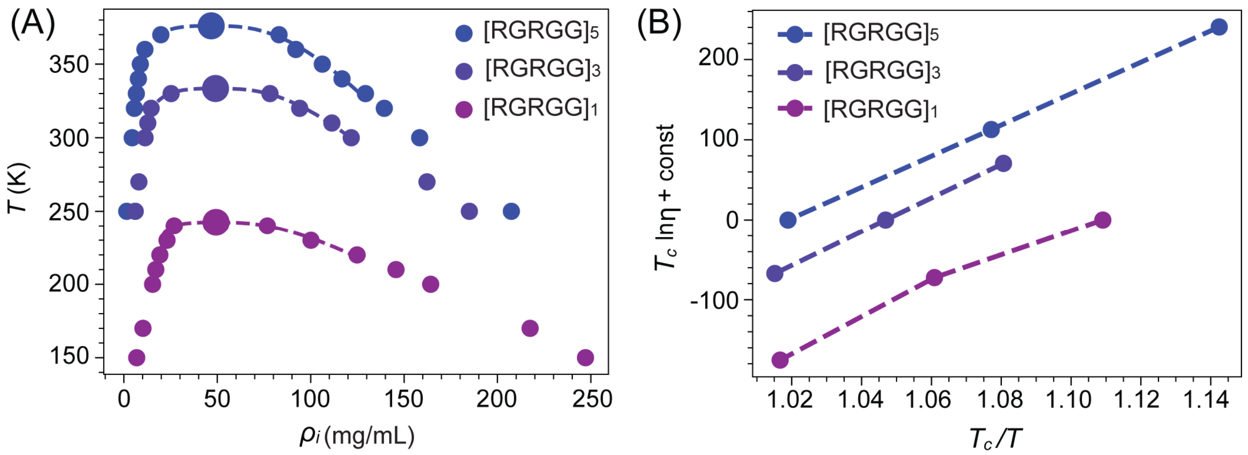

Figure S14. Effect of peptide repeat length on the phase diagram and activation energy of condensates obtained through coarse-grained simulations. (**A**) Phase diagram of [RGRGG]n-ssDNA condensates with n = 1, 3, and 5 in the space of temperature and biomolecular density obtained from direct coexistence simulations. (**B**) Arrhenius plots for different peptide chain lengths of RGRGG extracted from the temperature-dependent viscosity profiles.

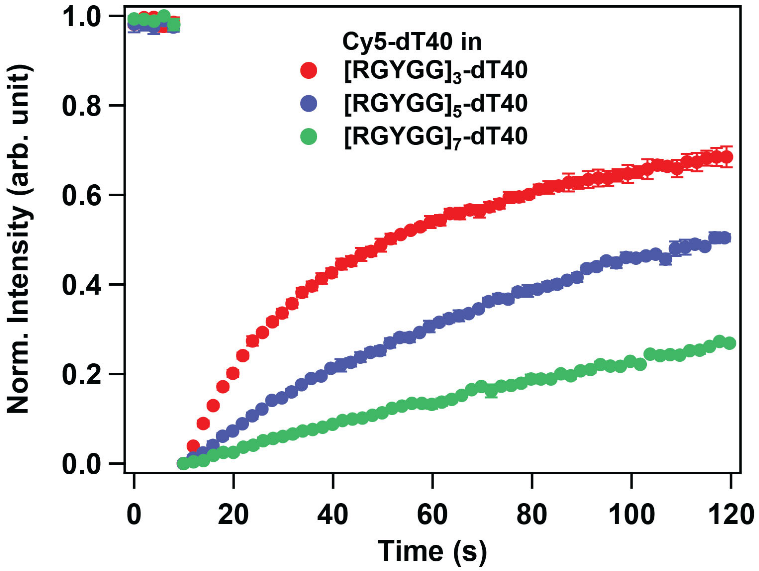

Figure S15. Effect of peptide repeat length on the mobility of dT40 in peptide-ssDNA condensates. Average FRAP recovery intensity traces for Cy5-labeled dT40 in condensates formed by $[RGYGG]_n$ for $n = 1$, 3, and 7 and dT40, respectively. Each time point is an average of 3-4 trials and the error is the standard deviation of the same.
